# Green synthesized silver nanoparticles mediated by *Fusarium nygamai* isolate AJTYC1: characterizations, antioxidant, antimicrobial, anticancer, and photocatalytic activities and cytogenetic effects

**DOI:** 10.1007/s11356-023-29414-8

**Published:** 2023-08-26

**Authors:** Abeer E. El-Ansary, Ahmed A. A. Omran, Heba I. Mohamed, Omima M. El-Mahdy

**Affiliations:** 1 Biochemistry Department, Faculty of Agriculture, Cairo University, Gamma St, Giza, 12613 Egypt; 2 Biological and Geological Sciences Department, Faculty of Education, Ain Shams University, Cairo, 11341 Egypt

**Keywords:** Antioxidants, Anticancer, Antimicrobial, Chromosome aberrations, Cytogenetic, Green synthesis, Photocatabylic

## Abstract

Green biosynthesized nanoparticles have a bright future because they can be produced using a method that is more energy-efficient, cost-effective, repeatable, and environmentally friendly than physical or chemical synthesis. In this study, silver nanoparticles (AgNPs) were produced using the *Fusarium nygamai* isolate AJTYC1. Several techniques were used to characterize the synthesized AgNPs, including UV–Vis spectroscopy, transmission electron microscope, zeta potential analysis, X-ray diffraction analysis, energy dispersive X-ray, and Fourier transform-infrared spectroscopy. AgNPs showed a distinctive surface plasmon resonance (SPR) peak in the UV–visible range at 310 nm. The morphology of the biosynthesized AgNPs was spherical, and the TEM image shows that they ranged in size from 27.3 to 53.1 nm. The notable peaks of the FT-IR results show the different groups for the alkane, alkynes, cyclic alkenes, carboxylic, aromatic amine, esters, and phenolics. Additionally, the results showed that AgNPs had superior antioxidant activity when compared to ascorbic acid and butylated hydroxytoluene, which is a powerful antioxidant. Additionally, AgNPs have antibacterial action utilizing agar diffusion against gram-positive bacteria, gram-negative bacteria, and antifungal activity. AgNPs’ anticancer activity varied depending on the type of cancer it was used to treat, including hepatocellular cancer (HepG2), colorectal carcinoma (HCT116), and breast cancer of the mammary gland (MCF7). The viability of the cancer cell lines was reduced with increasing AgNP concentration. AgNPs also demonstrated promising photocatalytic activity by reducing methylene blue, safranin, crystal violet, and green malachite by 88.3%, 81.5%, 76.4%, and 78.2%, respectively. In addition, AgNPs significantly affected the *Allium cepa* plant’s mitotic index and resulted in chromosomal abnormalities as compared to the control. Thus, the synthesized AgNPs demonstrated an efficient, eco-friendly, and sustainable method for decolorizing dyes as well as antioxidant, antibacterial, antifungal, and anticancer activities. This could be a huge victory in the fight against numerous dynamic diseases and lessen wastewater dye contamination.

## Introduction

Across the world, investigators and academics are drawn to the distinctive and inventive branch of science known as nanotechnology (Rudrappa et al. 2023). It is a new area of science that depends on the creation, identification, and use of tiny particles. These nanoparticles (NPs) are used in many different fields (Ihsan et al. 2023). In nanotechnology, particles with a size range of 1–100 nm are synthesized. Due to their high surface-to-volume ratio, compact size, catalytic activity, chemical stability, high conductivity, and biological characteristics, NPs display a variety of novel properties (Ranjani et al. 2021). Different methods have been used to create NPs from various metals. Silver-based NPs (AgNPs) have attracted the most interest among these due to their distinct properties and extensive use in a variety of fields, including biosensors, drug delivery, imaging, biomedicine, and agriculture (Mohamed et al. 2021). AgNPs have attracted some interest because of their distinct physicochemical and biological characteristics (Al-Rajhi et al. 2022; Dhawi et al. 2021).

The manufacturing of NPs can be accomplished using a variety of techniques, including chemical, physical, and biological ones. Chemical and physical approaches use harmful substances and produce dangerous byproducts that damage the environment (Moghaddam et al. 2015; Haris et al. 2023). The process for producing biological nanoparticles uses organisms like bacteria, actinomycetes, fungus, algae, and plants (Pandit et al. 2022). Compared to the chemical and physical approaches, the biological method, or green synthesis of NPs, is more efficient, environmentally benign, more widely available, and less expensive (Seetharaman et al. 2021). NPs can be produced by fungi, plants, and microorganisms through extracellular and intracellular processes. In order to defend against harmful substances, fungi, plants, and microorganisms operate as reducing agents, which change the chemical compositions of metals (Guliger-Casagrande et al. 2019; Mogazy et al. 2022; Rudrappa et al. 2023). Fungi are widely used among many biological agents because they are inexpensive, produce a lot of biomass, produce little toxicity, and use little energy (Ameen et al. 2023). Myconanotechnology is the process for creating NPs from fungi (Rudrappa et al. 2023). Extracellular proteins, enzymes, and metabolites are all abundantly produced by fungi. These biomolecules function as reducing agents but also contribute to NP capping, hence influencing the size and stability of NPs (Rai et al. 2021). Many researchers have chosen the genus *Fusarium* out of the various fungal genera employed for the manufacture of nanoparticles (Ingle et al. 2009). The use of *Fusarium* spp. for the production of nanoparticles has several benefits, including rapid growth, ease of cultivation, bulk extracellular production, scalability, cheap biomass handling, safety, and simple processing (Rai et al. 2021).

Silver nanoparticles (AgNPs) have generated a great deal of attention due to their distinct physical, optical, chemical, and biological features (Mohamed et al. 2021). For the production of AgNPs, the researchers utilized herbal extracts, fungi, bacteria, and algae (Sivasubramanian et al. 2023). The bioengineered AgNPs have numerous biological and biomedical uses. Surprisingly, the biogenic nanosized silver particles showed notable, antioxidant, antimicrobial, and anticancer activities (Alduraihem et al. 2023; Khodeer et al. 2023). Also, AgNPs are widely employed in tissue engineering, diagnostics, and drug delivery because of their distinctive surface area modification, characterization, ease of synthesis, chemical stability, low cost, optoelectronic, and physicochemical features (Hublikar et al. 2023). The phytochemicals found in several plant species can decrease Ag^+^ to Ag^0^ without the use of a microbial cell culture.

Antibiotic resistance (AR) in microorganisms has risen over the past few decades as a result of the uncontrolled and over-the-counter usage of antibiotics (Meena et al. 2020). As a result, the need for alternatives to antibiotics has grown recently (Bapat et al. 2022). Numerous researches nowadays have revealed that AgNPs are effective antibiotic substitutes because of their distinct characteristics, including their wide surface area, high energy, and smoothness (Dinesh et al. 2022). Many secondary metabolites, including flavonoids, phenols, terpenes, and proteins found in plants and microbes, contribute to this environmentally friendly one-step non-hazardous process for synthesizing AgNPs (Casagrande and Lima 2019).

Another major public health issue that is recognized globally is cancer. Because of coronavirus illnesses, there was a notable decrease in the detection and treatment of cancer in 2020 (Siegel et al. 2021). In this instance, it can result in unfavorable outcomes like higher mortality and advanced disease. For the majority of cancer patients, radiation therapy is frequently favored. However, negative consequences could include DNA damage and inhibition of cellular events occurring during cell division in normal cells (Baskar et al. 2012). Breast cancer is the most prevalent cancer and the second leading cause of cancer death in women. Thirty percent of breast cancer cases among other malignancies are recorded each year in females. Women in their middle to late years are most commonly diagnosed with breast cancer. The majority of tumors react to treatment initially, but with time, many develop resistance (Gurunathan et al. 2013). For cancer patients to receive effective treatment, innovative methods must be used. Studies relating to the usage of nanoparticles created through various processes in medicine (diagnosis and treatment) have come to light recently. These studies demonstrate the widespread usage of nanoparticles in the treatment of cancer (Almalki and Khalifa 2020; Gomathi et al. 2020).

Several industries, including textile, leather, plastic, paper, paint, cosmetics, and medicines, potentially use toxic organic dyes (Githala et al. 2022). Serious health issues such as problems with the central nervous system, eyes, and mental health might result from direct contact with the dye with organisms. An important environmental concern is the sequestration of synthetic organic molecules from wastewater (Varadavenkatesan et al. 2020). Physical and chemical techniques were not sufficient to completely remove synthetic dyes from wastewater, and they are expensive and energy-intensive; more advanced methods are required (Bonigala et al. 2018). Using metallic nanoparticles to remove harmful organic contaminants from wastewater is a novel method that offers an effective substitute for traditional physical and chemical methods (Eisa et al. 2019). Among the many metallic NPs, AgNPs have received the most research attention due to their relatively inexpensive cost (Githala et al. 2022).

The *F. nygamai* isolate AJTYC1 was used in the current investigation to biosynthesize AgNPs using an environmentally friendly and sustainable approach. the study also aimed to study the roles of green biosynthesized AgNPs as antioxidants, antibacterial, antifungal, anticancer, and photocatalytic characteristics, as well as their effects on chromosomal abnormalities and cytotoxicity in *Allium cepa* roots. The use of the *F. nygamai* isolate AJTYC1 in the biosynthesis of AgNPs is considered the first report.

## Materials and methods

### Materials

The study’s source of silver nitrate (AgNO_3_) was Sigma-Aldrich in Louis, Missouri, USA. The Modern Lab Co. in India provided the other grade chemicals, culture media, and reagents utilized in this work.

### Fungus used

**Fig. 1:**
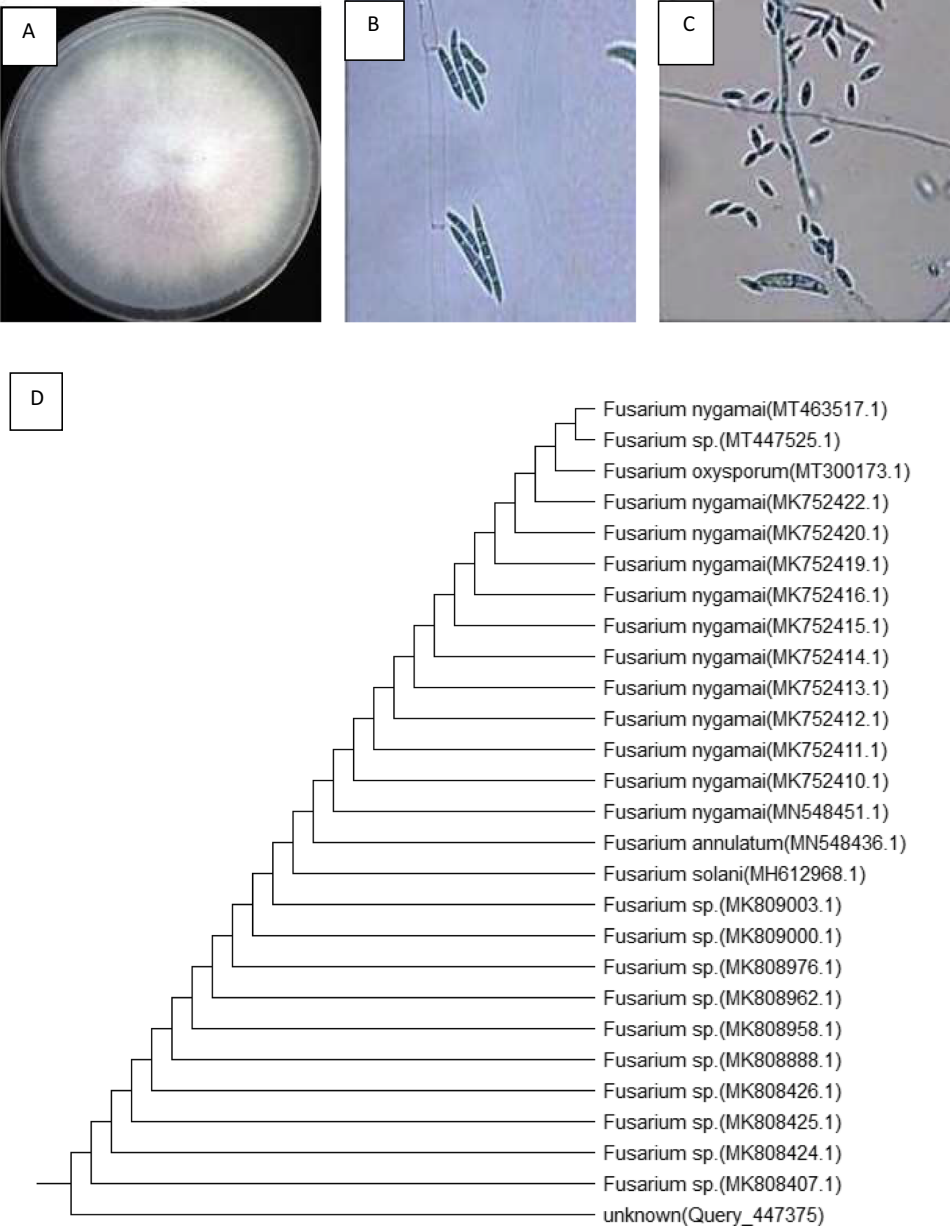
Morphological (**A**, **B**, **C**) and molecular identification (**D**) of *Fusarium nygamai* isolate AJTYC1

Silver nanoparticles were myco-synthesized using a biomass filtrate of *F. nygamai* isolate AJTYC1. The fungus was identified at the Faculty of Science, Botany Department, Assiut University. Based on the mycelium and spore morphology studies as shown in Fig. 1 A–C, this isolate was identified as *Fusarium sp.* and confirmed by the 18S rRNA gene. Using the 18S rRNA gene sequence and 100% homology of the isolate, the findings revealed a striking similarity to *F*. *nygamai* isolate AJTYC1. Using the nearby technique, the phylogenetic analysis and tree were constructed (Fig. 1D). The isolated strain was identified as *F. nygamai* isolate AJTYC1 based on DNA sequence analysis, and it was deposited in GenBank with the accession number MT463517.

### Biosynthesis of AgNPs using *F. nygamai* isolate AJTYC1 extract

Silver nanoparticles were produced using *F. nygamai* isolate AJTYC1 according to the method of Chandrasekhar and Vinay (2017). To prevent contamination, 10-day-old fungal mycelia biomass was rinsed in sterile, twice-distilled water. The extract was made by macerating fungal biomass in a 2:1 ratio with sterilized, double-distilled water. Using Whatman No. 1 filter paper, the aqueous solution was separated. For bioreduction, 90 mL of 5 mM silver nitrate was added to 10 mL of fungal extract and stirred continuously magnetically for 20 min. The reaction mixture was left to finish its reaction and bioreduction process overnight at room temperature (25 ± 2 °C) in a dark area.

### Characterization of AgNPs

#### Analysis of UV–visible spectra for AgNPs

By employing a UV–visible spectrometer for spectral analysis, the produced AgNPs were verified. Shimadzu UV–visible spectrophotometer model UV3600 was used to analyze the production of AgNPs in the 300–700 nm wavelength range. Centrifugation was used to separate the synthesized, purified AgNPs for 15 min at 15,000 rpm. AgNP-containing pellets were cleaned with Milli-Q water that had been sterilized. A hot air oven was used to dry the pellet for 24 h at 50 °C, and the pure powder was used for the other steps of characterization.

#### Transmission electron microscopy (TEM)

Following the procedures outlined by Kumar et al. (2019), the particle shape and size of the produced NPs were further validated by high-resolution TEM (JEM-100XX). The diameter of the NPs was measured in order to establish the average size of the particles.

#### Scanning electron microscopy (SEM) Analysis

AgNPs’ size, shape, distribution, and composition were investigated using SEM. A drop of AgNPs was applied to a copper grid that had carbon coating, and the sample was dried using a mercury lamp for 5 min before measurements at various magnifications were taken.

#### X-ray diffraction (XRD) analysis

The produced AgNPs were homogenized and finely milled to achieve a bulk composition of identical size (Dimitrijevic et al. 2013). XRD was used to study the materials’ crystalline structure at room temperature using a PW-3710 X’pert Philips diffractometer with Cu Kα radiation (*λ* = 1.54060 A), 40 mA, 40 kV, and a 2.5°/min scan rate.

#### Zeta potential analysis using DLS

DLS was used to examine the stability and refractive index of AgNPs in order to determine the zeta potential (Amaladhas et al. 2012). To quantify the zeta potential, 2000 µL of AgNPs were transferred to clear disposable zeta cells. The zeta potential was determined using dynamic light scattering with a scattering angle of 90 °C and a laser wavelength of 780 nm at a temperature of 25 °C. The stability and size distribution of the nanoparticles can be determined with the aid of this method.

#### Energy dispersive X-ray (EDX) analysis

SEM coupled with an EDX analyzer (JEOL, JSM-6380 LA, Tokyo, Japan) was used to conduct an elemental study of AgNPs.

#### Fourier transformed infrared spectroscopy analysis

The FT-IR was employed to capture the functional groups bound to AgNPs. Using KBr pellets, the ground-up pellet mixture was resuspended in 1 mL of deionized water. A drop of the reaction was used for the FT-IR analysis (Govindappa et al. 2018).

### Antioxidant properties

#### Free radical scavenging activity

Using the method described by Niknezhad et al. (2018), AgNPs antioxidant activity was evaluated. To produce final concentrations of 50–200 µg/mL of AgNPs, the stock ethanolic solution of the nanoparticles was diluted with ethanol. One milliliter of 0.2 mM DPPH was then added, and the mixture was continuously stirred for the following 30 min at room temperature. Using a spectrophotometer, the optical density of the solution was measured at 517 nm.

$$\%\;\mathrm D\mathrm P\mathrm P\mathrm H=\frac{\mathrm{Absorbance}\;\mathrm{of}\;\mathrm{control}-\mathrm{Absorbance}\;\mathrm{of}\;\mathrm{sample}}{\mathrm{Absorbance}\;\mathrm{of}\;\mathrm{control}}\times100.$$

To calculate the *IC*_50_ value, the percentage of radical scavenging activity was displayed against the appropriate AgNP concentration. The *IC*_50_ value is the greatest concentration of an ingredient required to reduce it by 50%.

#### ABTS radical cation decolorization assay

With the use of a modified version of Li et al. (2012), the ABTS test was carried out. Five milliliters of a 4.9 mM potassium persulfate solution and a 7 mM ABTS solution were mixed. The combination was added to 0.2 mL of various ethanolic extract concentrations of AgNPs (varying from 50 to 200 µg/mL) and 1.8 mL of ABTS reagents after 16 h of dark storage at room temperature. At 734 nm, the mixture’s absorbance was measured. L-ascorbic acid 0.3 mM was used as a control.

$$\%\;\mathrm I\mathrm n\mathrm h\mathrm i\mathrm b\mathrm i\mathrm t\mathrm i\mathrm o\mathrm n=\frac{\mathrm{ABS}\;\mathrm{control}\;-\;\mathrm{ABS}\;\mathrm{sample}}{\mathrm{ABS}\;\mathrm{control}}\times100$$

#### Determination of hydroxyl radical (OH) scavenging activity

AgNPs’ ability to scavenge OH radicals was determined using Ye et al. (2012) protocol. One milliliter of phosphate buffer saline (0.15 mM, pH 7.4), 1 mL of EDTA-Fe(II) (6 mM), and 8 mL of H_2_O_2_ (6%, v/v) were combined with 0.7 mL of AgNPs concentrations before being incubated at 40 °C for 30 min. The absorbance was read after incubation with a spectrophotometer at 520 nm. The fluctuation in absorbance of the reaction mixture served as a gauge of AgNPs’ ability to scavenge hydroxyl radicals. Ascorbic acid served as the standard. The following equation was used to calculate the hydroxyl radical’s scavenging capacity:

$$\%\;\mathrm s\mathrm c\mathrm a\mathrm v\mathrm e\mathrm n\mathrm g\mathrm i\mathrm n\mathrm g\;\mathrm a\mathrm c\mathrm t\mathrm i\mathrm v\mathrm i\mathrm t\mathrm y=\frac{\mathrm{Sample}\;\mathrm{with}\;\mathrm{reagent}-\mathrm A\;\mathrm{blank}}{\mathrm{Reagent}\;\mathrm{with}\;\mathrm H2\mathrm O2-\mathrm{Reagent}\;\mathrm{only}}\times100.$$

#### Hydrogen peroxide scavenging assay

AgNP concentrations of 50, 100, 150, and 200 µg/mL were employed for the hydrogen peroxide scavenging activity, with ascorbic acid serving as the standard (Bhakya et al. 2016). Separately, 50 μL of a 5 mM H_2_O_2_ solution and various doses of AgNPs and ascorbic acid were combined. The reaction mixture was then incubated for 20 min at room temperature (25 ± 2 °C). Based on the equation above, the examined reaction mixture’s absorbance at 610 nm and its H_2_O_2_ scavenging percentage was computed.

#### Assay of reducing power

Twenty-five milliliters of 200 mM phosphate buffer (pH 6.6) and 2.5 mL of 1% potassium ferric cyanide each received a different amount of AgNPs at various concentrations. After 20 min of 20 °C incubation, the reaction mixture was promptly cooled. Centrifuging the reaction mixture for 8 min at 3000 rpm with 2.5 mL of 10% TCA was added after incubation. Millipore Milli-Q water was distributed equally among everyone. After that, the upper layer added 1 mL of ferric chloride at a concentration of 0.1%, and the absorbance at 700 nm was measured. With the use of the aforementioned equation, the results were compared using conventional BHT, and the decreasing power percentage was computed.

#### Antimicrobial activity of AgNPs

AgNPs antimicrobial activity was assessed using gram-positive bacteria (*Bacillus subtilis*, *Staphylococcus aureus*), gram-negative bacteria (*Pseudomonas aeruginosa*, *Escherichia coli*), and fungi (*Aspergillus niger, Candida albicans*). The well diffusion method was employed to evaluate antimicrobial activity (El-Beltagi et al. 2022). The inhibitory zones were determined by placing 100 µL of the crude combination into separate 6-mm-diameter wells cut in nutritional agar plates that had been inoculated with test bacteria and potato dextrose agar (PDA) plates that had been inoculated with test fungus. The diameter of the zone of inhibition was then measured in millimeters after incubation at 37 °C for 24 h for the bacteria-containing plates and 72 h for the fungi-containing plates. The average of the three results from each test was calculated.

#### Anticancer activity of AgNPs

Three distinct cell lines were used in this investigation, including hepatocellular cancer (HepG2), breast cancer of the mammary gland (MCF7), and colorectal carcinoma colon cancer (HCT116). The cell lines were obtained and activated by VACSERA-Cell Culture Unit, Cairo, Egypt, in conformity with the ethical guidelines of the scientific community.

A full monolayer sheet developed in the 96-well tissue culture plate after 24 h of incubation at 37 °C with 1 × 10^5^ cells/mL (100 µL/well). Three wells served as controls and received nothing more than maintenance media whereas several wells received 0.1 mL of each dilution for testing. The plate was tested after a 37 °C incubation period. The physical characteristics of toxicity were examined in the cells. Twenty microliters of the MTT (5 mg/mL in PBS) solution were given to each well. Shake the media vigorously at 150 rpm for 5 min to completely absorb the MTT. The MTT was incubated for 1 to 5 h at 37 °C with 5% CO_2_ to allow for digestion. Wipe the media clean if any residue has to be eliminated, and then dry the plate with paper towels. In order to resuspend formazan, an MTT metabolic product, 200 µL of DMSO should be utilized. Shake the formazan and solvent in a shaker at 150 rpm for 5 min to completely mix them. In accordance with the technique of Wilson (2000), the absorbance was measured at 560 nm and subtracted at 620 nm. The following formula was used to assess the cell viability:

$$\%\;\mathrm R\mathrm C\mathrm V=\frac{\mathrm A570\;\mathrm{of}\;\mathrm{treated}\;\mathrm{samples}}{\mathrm A570\;\mathrm{of}\;\mathrm{untreated}\;\mathrm{sample}}\times100$$

#### Dyes decolorization processes by AgNPs

Batch tests were conducted on the sequestration of the dyes methylene blue, safranin, crystal violet, and green malachite in a 250-mL conical flask containing 50 mL of dye solution. The effects of the initial dye concentration were investigated (10–100 mg/L). Methylene blue, safranin, crystal violet, and green malachite dye solution were combined, and a required amount (0.1 g) of AgNPs was added. The mixture was then agitated at 200 rpm for an equilibrium period with a magnetic stirrer at room temperature. The solution was filtered to distinguish pellet and supernatant after the equilibrium period (Ramalingam et al. 2015). Using a UV–Vis spectrophotometer with a maximum 660 nm wavelength, the collected supernatant was examined to determine the concentrations of the remaining solution’s methylene blue, safranin, crystal violet, and green malachite dyes. The following equation has been used to calculate the decolorization efficiency (%):

$$\mathrm D\%=\frac{\mathrm{The}\;\mathrm{initial}\;\mathrm{concentration}\;\mathrm{of}\;\mathrm{dye}\;(\mathrm{Start}\;\mathrm{absorbance}\;-\;\mathrm{The}\;\mathrm{Concentration}\;\mathrm{of}\;\mathrm{dye}\;\mathrm{after}\;\mathrm{irradiation}\;(\mathrm{end}\;\mathrm{absorbance}}{\mathrm{The}\;\mathrm{initial}\;\mathrm{concentration}\;\mathrm{of}\;\mathrm{dye}}\times100$$

### Nano-toxicity assay of AgNPs

#### *A. cepa* test and treatment

Just prior to usage, experimental concentrations were made by diluting a stock solution of AgNPs (1000 mg/L) in distilled water and then sonicating the mixture for 30 min at 320 W and 35 kHz. Agricultural Research Centre, Giza, Egypt’s Field Crops Research Institute provided healthy onion bulbs (*A. cepa* L., 2*n* = 16) for this study. In 50 cm^3^ beakers, triplicates of bulbs with an average weight of 25 g were cultured for 48 h at room temperature (26 ± 2 °C), with distilled water being replaced twice daily. The test was conducted using freshly emerging roots that were 1.5 cm long and a treated solution in place of distilled water. In the absence of direct light, onion roots were treated with a range of AgNPs concentrations (10, 20, and 30 mg/L) for three different exposure durations (3, 6, and 12 h). Distilled water was used to treat the control groups. Samples of root tips were taken and washed thoroughly with distilled water.

### Cytological analysis

Washed tips from each treatment were fixed in a 3:1 (v/v) ethanol/glacial acetic acid mixture for 24 h and finally stored at 4 °C in a 70% ethanol solution. Fixed root tips were hydrolyzed in 1 N HCl at 60 °C for 10 min right before examination, and the hydrolysis solution was then replaced by Feulgen stain for around 3–4 h. After being cut and squashed between a slide and a cover in a drop of 45% acetic acid, the heavily stained meristematic region was studied under a light microscope at a magnification of 1000 × magnification. A total of 5000 cells from the control and each concentration of treatment were scored. By dividing the number of dividing cells by the total number of cells examined, the mitotic index (M.I.) was calculated as a percentage, and also, the percentages of mitotic phases were scored. In each stage, abnormalities were counted and expressed as a proportion of dividing cells.

### Statistical analysis

The means of three independent replicates were used to calculate all of the results in this study. The data variance was examined using SPSS v17 statistical software. Duncan’s multiple range test was used to analyze the mean difference between the treatments at a significant level of *p* ≤ 0.05.

## Results

### Synthesis and characterization of silver nanoparticles

*F. nygamai* isolate AJTYC1 was used to green synthesize AgNPs (Fig. 1). The color change that occurs during the chemical reactions in this method serves to identify the production of AgNPs. The reduction of silver ions (Ag^1+^) to zero-valent silver particles (Ag^0^) is indicated in our case by the color change from light yellow to a yellowish-brown color.

**Fig. 2:**
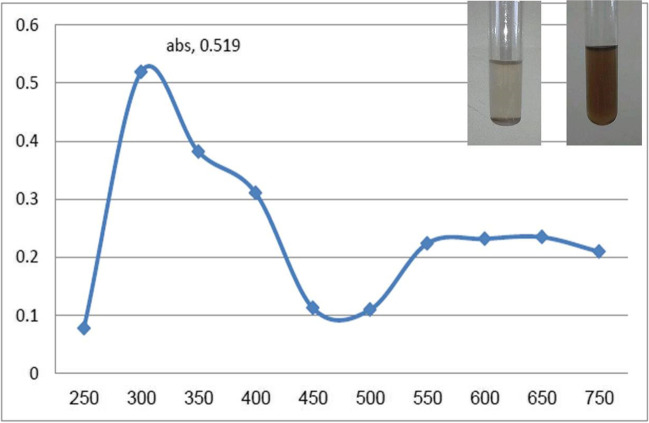
UV–Vis absorption spectra of green synthesized AgNPs using *F. nygamai* isolate AJTYC1 extract

Due to the presence of certain secondary metabolites, *F. nygamai* isolate AJTYC1 extract has a dual function, acting as both a reducing and stabilizing agent. UV–Vis spectroscopy results also supported the production of the biosynthesized AgNPs. A large absorption band of about 420 nm in Fig. 2 is indicative of the production of AgNPs. Additionally, a shoulder in the UV–Vis spectra at 310 nm is connected to the creation of Ag nanoclusters. This beak, which is caused by the surface plasmon resonance characteristic of AgNPs, shows that the particles were evenly distributed without aggregating.

**Fig. 3:**
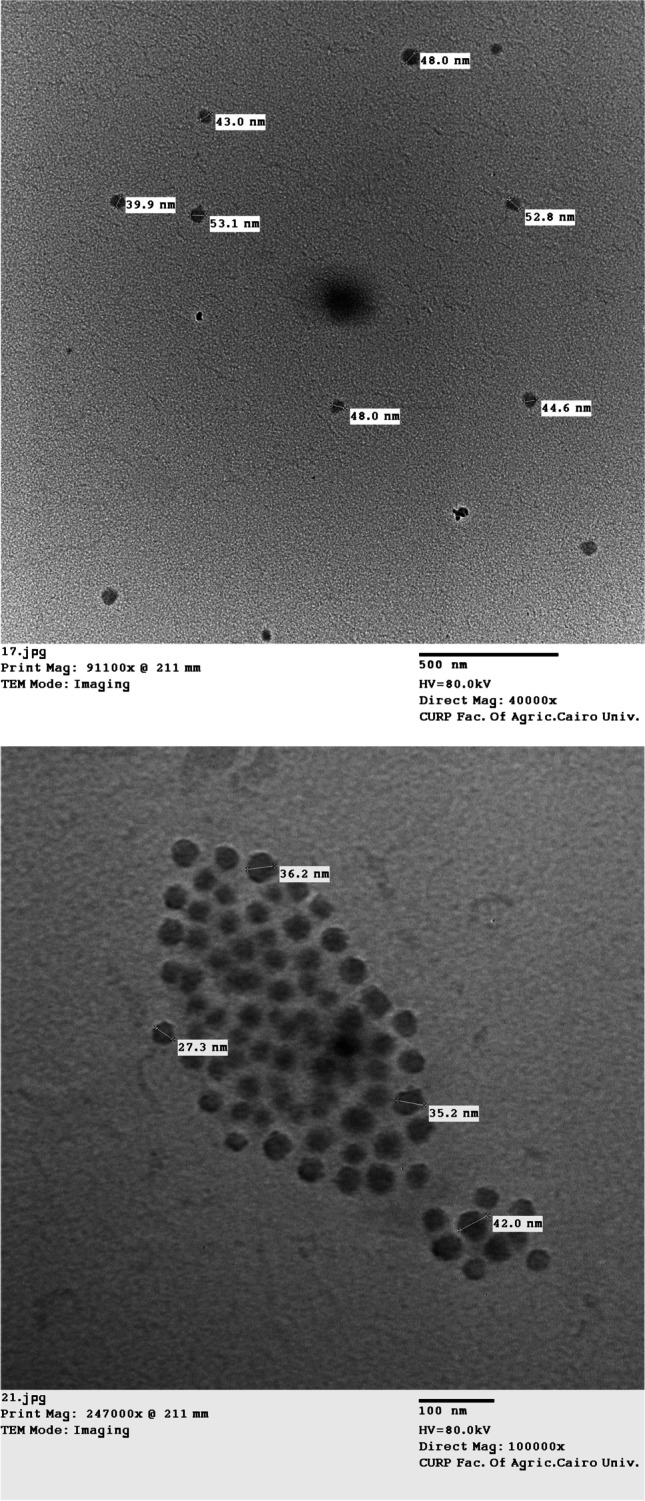
TEM micrographs of green synthesized AgNPs using *F. nygamai* isolate AJTYC1 extract

**Fig. 4:**
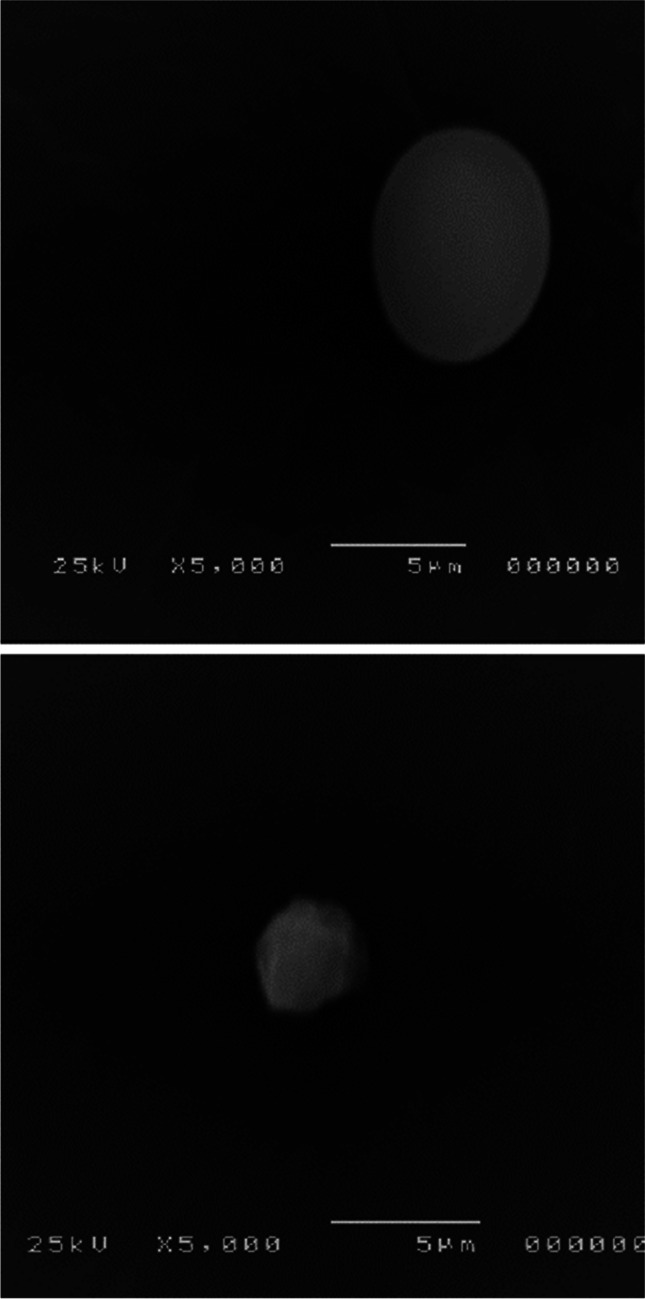
SEM micrographs of green synthesized AgNPs using *F. nygamai* isolate AJTYC1 extract

Using TEM and SEM, as depicted in Figs. 3 and 4, the produced AgNPs’ morphological and structural characteristics were verified. AgNPs are depicted in TEM micrographs as being spherical and free of any aggregation. According to Fig. 3, the particle size ranged from 27.3 to 53.1 nm.

**Fig. 5:**
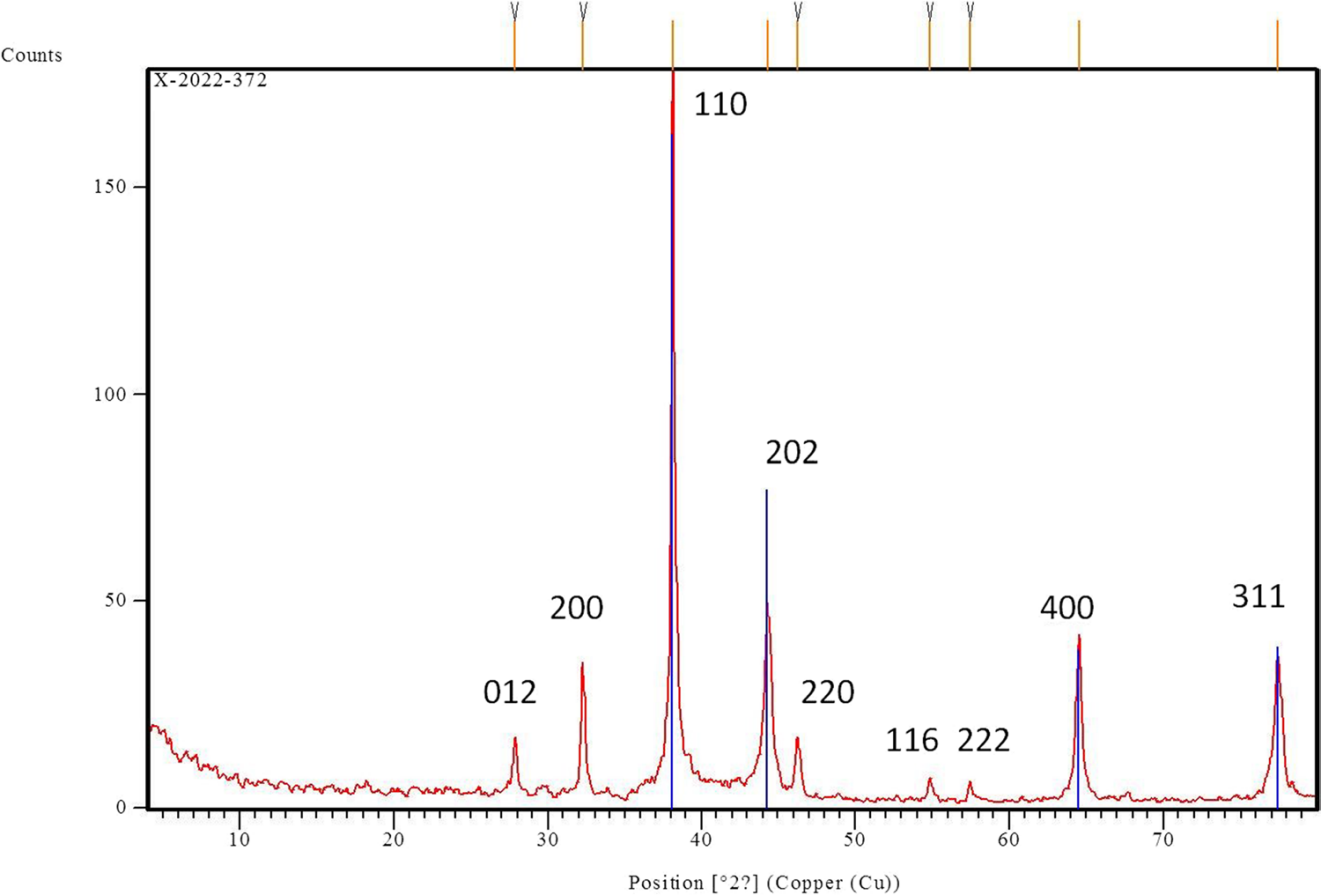
XRD patterns of green synthesized AgNPs using *F. nygamai* isolate AJTYC1 extract

In order to confirm the AgNPs particle’s crystallinity, additional analysis employing X-ray diffraction was conducted. As seen in Fig. 5, XRD-based AgNPs characterization exhibits nine peaks at 2θ values 27.8°, 32.3°, 38.2°, 44.4°, 46.2°, 54.9°, 57.5°, 64.5°, and 77.4° which assigned to planes 012, 200, 110, 202, 220, 116, 222, 400, and 311, respectively, for AgNPs.

**Fig. 6:**
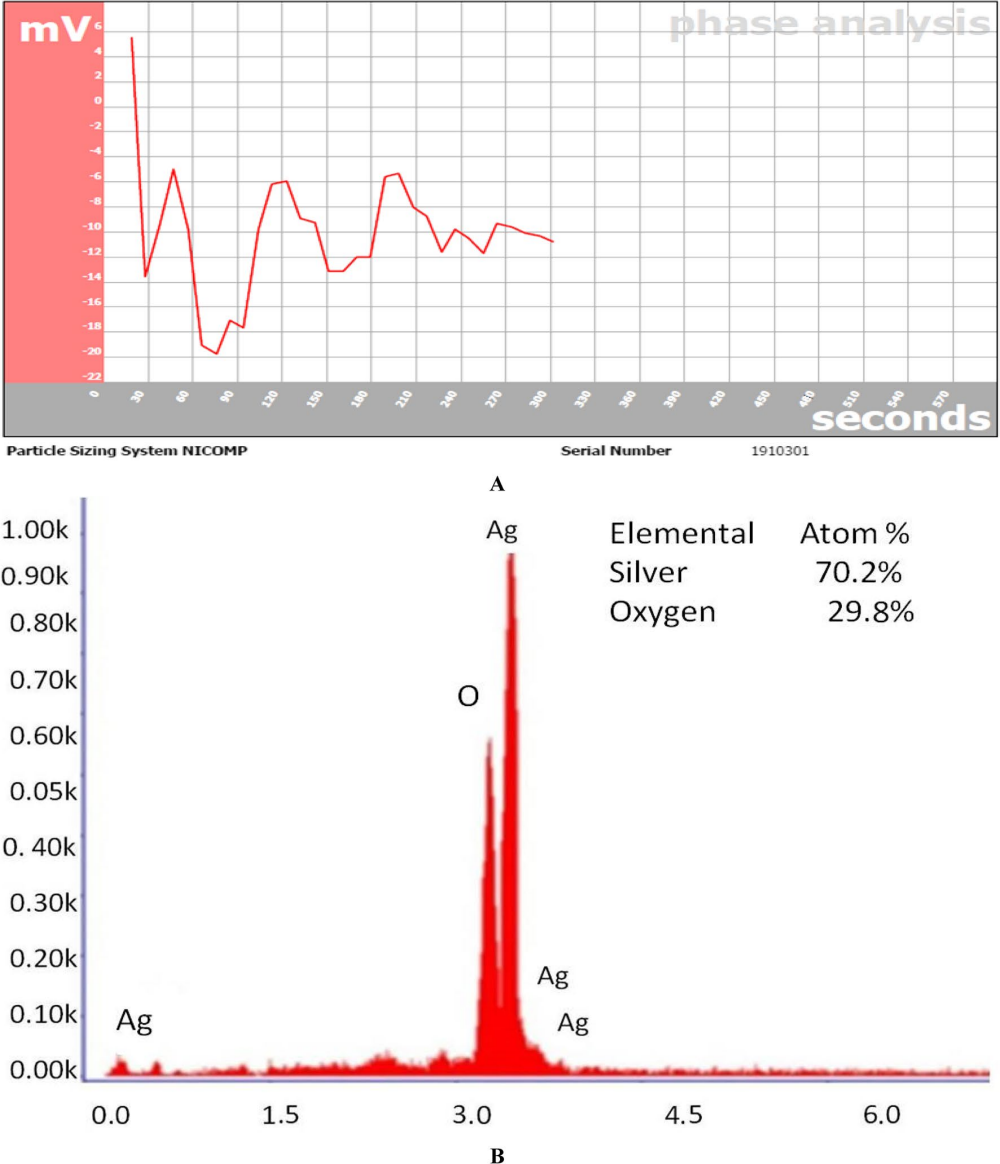
**A** Zeta-potential data and **B** energy dispersive spectroscopy (EDS) analysis for green synthesized AgNPs using *F. nygamai* isolate AJTYC1 extract

By using *F. nygamai* extract, produced AgNPs had a zeta-potential of approximately − 10.79 mV (Fig. 6A). The nanoparticles’ stability was demonstrated by the negative value, which prevented nanoparticle aggregation. The elemental mapping of the biogenic AgNPs was discovered using an energy-dispersive X-ray spectroscopy (EDX) study. According to Fig. 6B, an EDX examination revealed the presence of silver. At 3.2 keV, a high signal of the peak was seen, which is typical for metallic silver nanoparticle absorption.

**Fig. 7:**
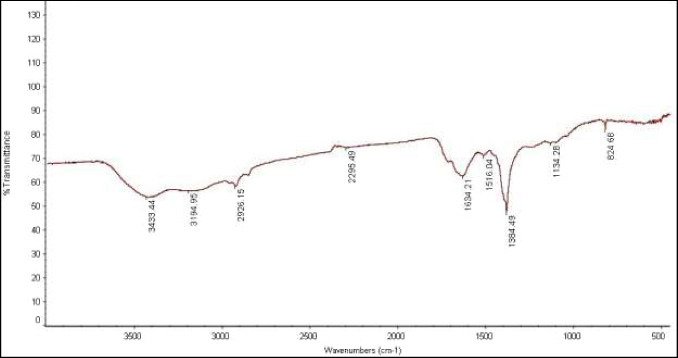
FT-IR spectra for green synthesized AgNPs using *F. nygamai* isolate AJTYC1 extract

The potential biomolecules responsible for the stabilization of the synthesized AgNPs were found using the FT-IR data. FT-IR analysis was used to look into the different functional groups in responsible for the reduction and stability of AgNPs. AgNPs synthesized from *F. nygamai* were FT-IR analyzed to find vibrational frequencies at 3433.4, 3194.9, 2926.2, 2295.5, 1634.2, 1516.04, 1384.5, 1134.3, and 824.7 cm^−1^ as shown in Table 1 and Fig. 7.

**Table 1:** FT-IR spectra of AgNP showing different peaks corresponded with functional groups attached

Absorption Peaks (cm^−1^)	Bond	Functional agents
3433.44	O–H stretching	Phenolics
3194.95	O–H stretching, N—H stretch	Alcohols and amides
2926.15	C-H stretching	Alkane
2295.49	C = C stretching	Alkynes
1634.21	C = C stretching	Cyclic alkenes
1516.04	C = O	carboxylic group
1384.49	C-N stretching	Aromatic amine
1134.28	C—O stretching	Esters
824.68	C = C bending	Alkene

### Antioxidant activity of AgNPs

Antioxidant influence on DPPH radical scavenging, ABTS, and hydroxyl radical scavenging was linked to their capacity to donate hydrogen (Table 2). DPPH is a stable free radical that can be converted into a stable molecule by the addition of an electron or hydrogen radical. In contrast to ascorbic acid and butylated hydroxytoluene, which were used as controls, Table 2 demonstrates that scavenging of DPPH radicals, ABTS, OH, H_2_O_2_, and reducing power increased with increasing the concentrations of AgNPs produced from *F. nygamai* (50, 100, 150, and 200 µg/mL). The highest increases in DPPH radicals (66.91%), ABTS (49.1%), hydroxyl radical scavenging (53.0%), H_2_O_2_ (70.3%), and reducing power (65.7%) were observed at 200 µg/mL of AgNPs. Comparing the H_2_O_2_ scavenging activity of the AgNPs to that of DPPH, ABTS, and hydroxyl radicals revealed that they were more effective. In comparison to BHT (72.5%), the AgNPs demonstrated a 65.7% reducing power assay. The *IC*_50_ values of DPPH radicals, ABTS, OH scavenging, H_2_O_2_, and reducing power were 146.58, 203.67, 187.77, 131.87, and 152.91 µg/mL, respectively.

**Table 2:** Antioxidant activities (DPPH, ABTS, and OH scavenging) of AgNPs produced from *F. nygamai* isolate AJTYC1 as well as ascorbic acid and butylated hydroxytoluene at different concentrations

Treatment	Concentration (µg/mL)	% Inhibition of DPPH	*IC*_50_ (μg/mL)	% Inhibition of ABTS	*IC*_50_ (μg/mL)	% inhibition of OH	*IC*_50_ (µg/mL)	% Inhibition of H_2_O_2_	*IC*_50_ (µg/mL)	% Inhibition of reducing power	*IC*_50_ (µg/mL)
AgNPs	50	15.08 ± 1.0	146.58	10.01 ± 1.0	203.67	19.7 ± 0.5	187.77	25.02 ± 0.2	131.87	18.5 ± 0.1	152.91
	100	33.81 ± 1.0		27.35 ± 2.0		27.3 ± 0.7		40.9 ± 0.3		32.0 ± 0.2	
	150	54.02 ± 1.5		37.29 ± 2.5		42.3 ± 1.2		55.5 ± 0.5		48.5 ± 0.2	
	200	66.81 ± 2.0		49.10 ± 3.0		53.0 ± 1.5		70.3 ± 0.6		65.7 ± 0.5	
Ascorbic acid	5	22.5 ± 1.0	19.05	30.34 ± 1.0	23.7	30.0 ± 0.2	27.4	18. 1 ± 0.1	32.95	20.6 ± 0.2	19.92
	10	38.9 ± 1.5		33.89 ± 1.2		35.0 ± 0.3		35. 2 ± 0.3		35.3 ± 0.2	
	20	50.1 ± 1.6		47.93 ± 1.6		45.0 ± 0.4		40.4 ± 0.3		50.2 ± 0.3	
	40	70.2 ± 2.3		66.85 ± 1.9		60.3 ± 0.9		60.7 ± 0.4		70.3 ± 0.4	
Butylated hydroxytoluene (BHT)	5	22.1 ± 0.9	19.0	35.3 ± 1.2	11.8	20.3 ± 0.2	26.4	20.6 ± 0.2	39.76	22.8 ± 0.2	18.98
	10	35.3 ± 1.3		50.2 ± 1.4		30.6 ± 0.4		29.4 ± 0.2		36.9 ± 0.3	
	20	60.2 ± 1.8		65.9 ± 1.6		45.5 ± 0.5		40.2 ± 0.3		52.7 ± 0.3	
	40	80.5 ± 2.5		90.4 ± 2.2		65.4 ± 0.7		50.3 ± 0.3		72.5 ± 0.4	

The values are the means of three replicates with standard deviation (± SD)

### Antimicrobial activity of AgNPs

**Fig. 8:**
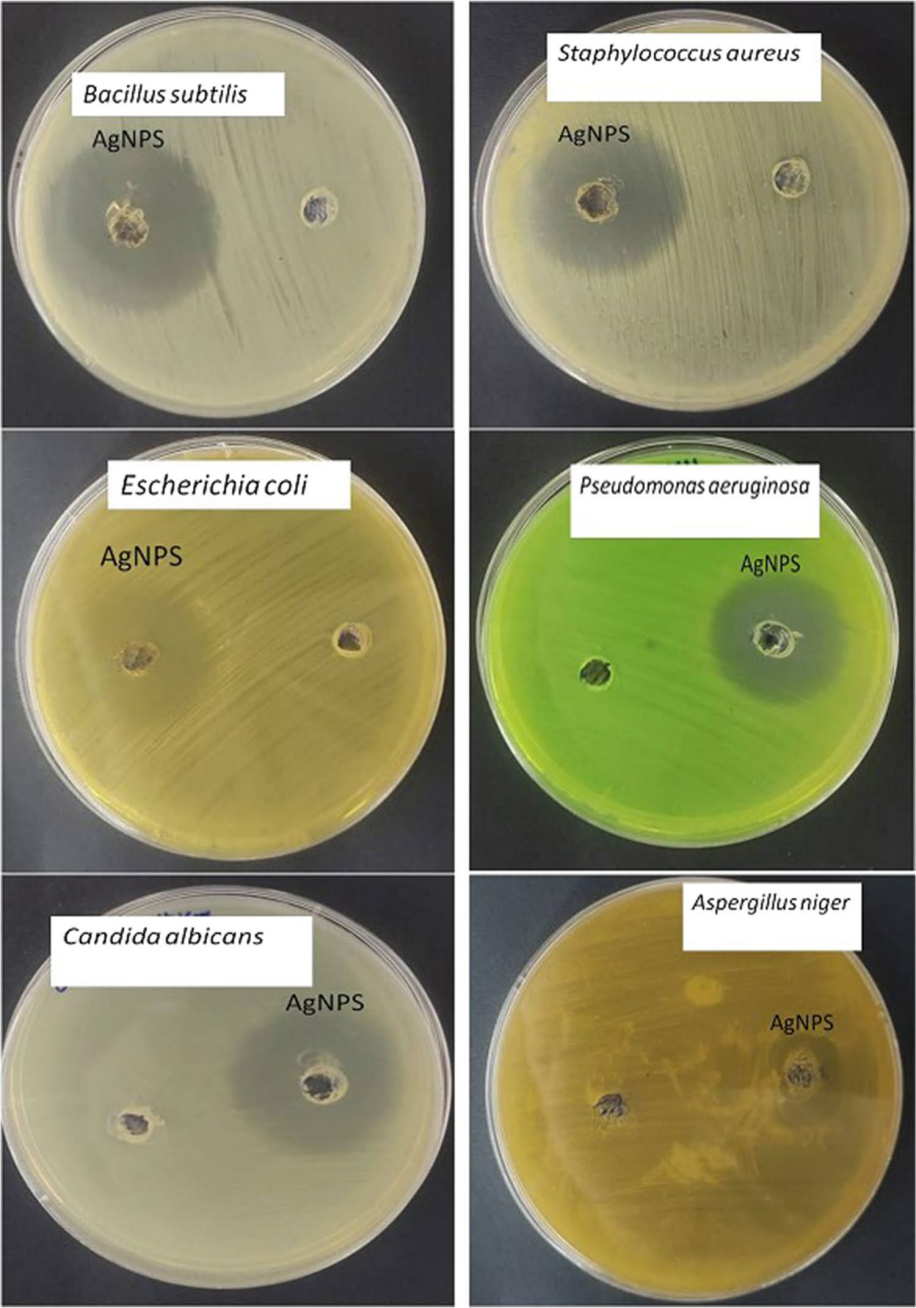
Antimicrobial efficiency of AgNPs produced from *F. nygamai* isolate AJTYC1 against gram-positive, gram-negative, and fungi

**Fig. 9:**
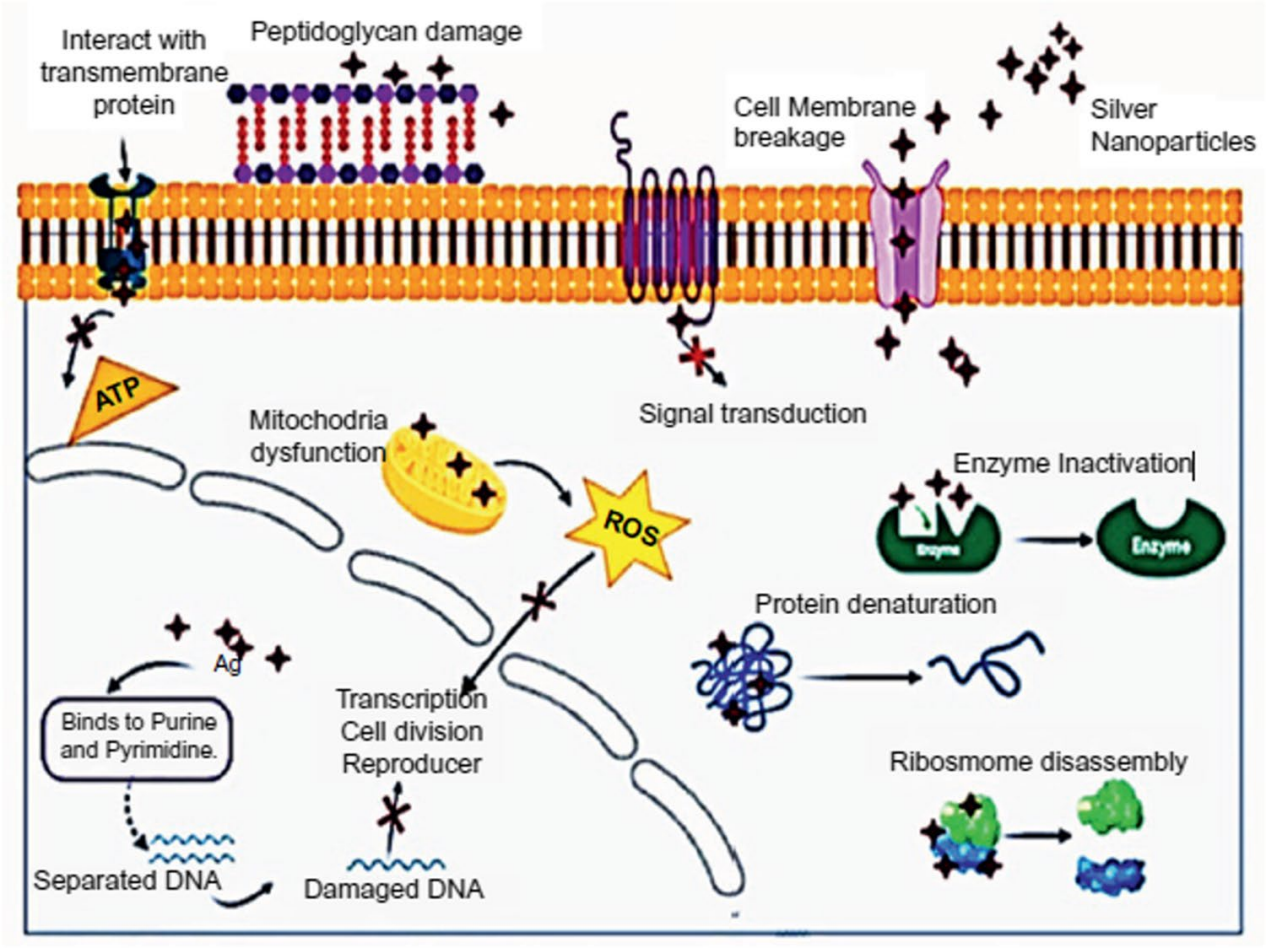
The mechanism of AgNPs on microbial cells

The data in Table 3 and Figs. 8 and 9 show the antimicrobial efficacy of AgNPs against a group of microbes that includes gram-positive bacteria, gram-negative bacteria, and fungi using the agar diffusion method. According to the research, AgNPs displayed antimicrobial efficacy against gram-positive, gram-negative, and fungi. The inhibitory zones of *P. aeruginosa*, *E. coli*, *S. aureus*, *B. subtilis*, *C. albicans*, and *A. niger* had sizes of approximately 24, 30, 22, 23, 33, and 20 mm, respectively.

**Table 3:** Antimicrobial efficiency of AgNPs produced from *F. nygamai* isolate AJTYC1 against gram-positive, gram-negative, and fungi

Pathogenic microorganism	Inhibitions zones (mm)	Con
Gram + ve bacteria		
*Bacillus subtilis* (ATCC 6633)	24 ± 0.02	19
*Staphylococcus aureus* (ATCC 6538)	30 ± 0.03	27
Gram − ve bacteria		
*Escherichia coli* (ATCC 8739)	22 ± 0.02	18
*Pseudomonas aeruginosa* (ATCC 90274)	23 ± 0.04	21
Fungi		
*Candida albicans* (ATCC 10221)	33 ± 0.05	28
*Aspergillus niger*	20 ± 0.02	15

The values are the means of three replicates with standard deviation (± SD)

### Anti-cancer activity of AgNPs

**Fig. 10:**
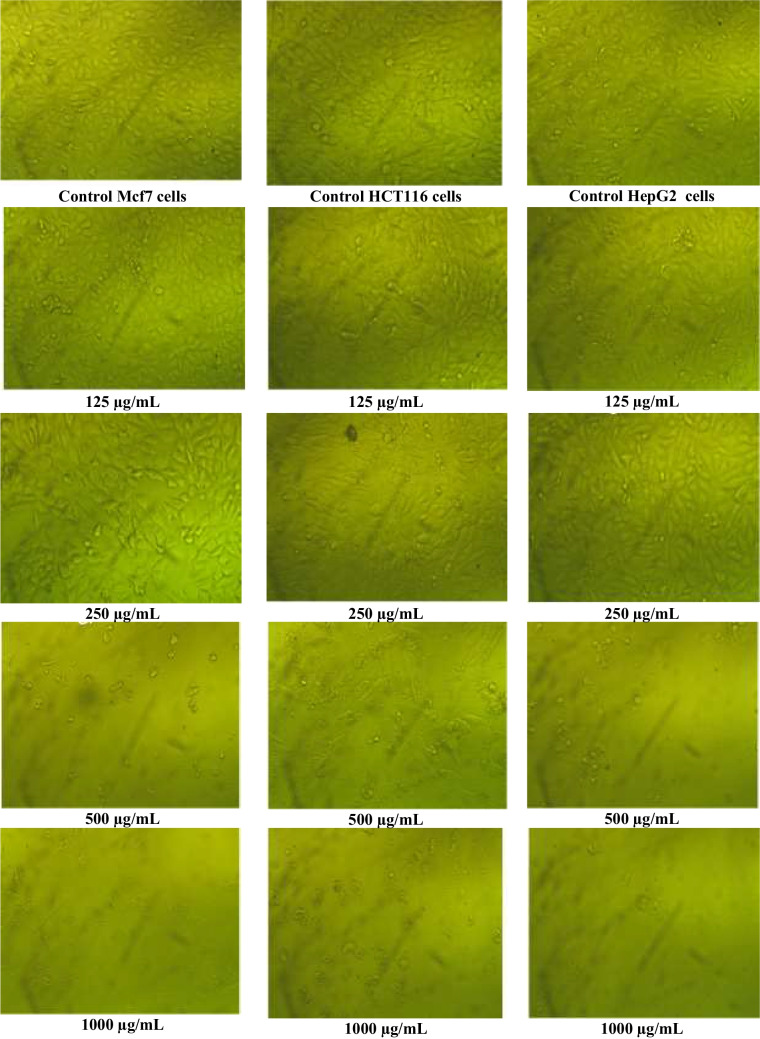
Effect of different concentrations of AgNPs produced from *F. nygamai* isolate AJTYC1 on MCF7, HCT116, and HepG2 cells

According to Table 4 and Fig. 10, the growth inhibitory effects of various AgNP doses (31.25, 62.5, 125, 250, 500, and 1000 µg/mL) against hepatocellular cancer (HepG2), colorectal carcinoma colon cancer (HCT116), and breast cancer of the mammary gland (MCF7) were investigated. The results showed that AgNPs exhibited varying degrees of anticancer action towards the cancer cell lines that were under investigation and that the viability of the cells reduced with increasing AgNPs concentration. As illustrated in Fig. 10, the morphological alterations of the cancer cells after treatment indicated a variety in size, blebbing of the cell membrane, deformed cells, cell shrinkage, and roundness to varied degrees. These morphological changes were shown in cancer cells treated with AgNPs compared to control. The most pronounced effects of AgNPs were detected at a dose of 1000 µg/mL, which inhibits the viability of MCF-7 (breast cancer) by about 2.49% with a toxicity of about 97.51%, the viability of HCT116 (colon cancer) by about 5.44% with a toxicity of 94.56%, and the viability of HepG2 (hepatocellular cancer) by about 2.67% with a toxicity of 97.33%. The *IC*_50_ for breast cancer was 302.93 µg/mL, colon cancer was 538.92 µg/mL, and hepatocellular cancer was 309.98 µg/mL.

**Table 4:** Effect of different concentrations of AgNPs produced from *F. nygamai* isolate AJTYC1 on MCF7, HCT116, and HepG2 cells

Cell line	Conc. of silver nanoparticles (µg/mL)	Mean O. D	ST. E	Viability %	Toxicity %	*IC*_50_ (µg/mL)
MCF7	Control	0.722	0.007	100	0	302.93
	1000	0.018	0.0006	2.49	97.51	
	500	0.010	0.008	13.80	86.20	
	250	0.342	0.008	47.323	52.68	
	125	0.715	0.005	99.08	0.92	
	62.5	0.720	0.006	99.77	0.23	
	31.25	0.721	0.0074	99.91	0.09	
HCT116	Control	0.680	0.012	100	0	538.92
	1000	0.037	0.008	5.44	94.56	
	500	0.244	0.019	35.83	64.17	
	250	0.651	0.010	95.69	4.31	
	125	0.676	0.010	99.81	0.20	
	62.5	0.678	0.003	99.75	0.25	
	31.25	0.680	0.006	100	0	
HepG2	Control	0.675	0.010	100	0	309.98
	1000	0.018	0.0006	2.67	97.33	
	500	0.063	0.026	9.33	90.67	
	250	0.412	0.009	62.17	37.83	
	125	0.667	0.008	98.771	1.23	
	62.5	0.667	0.006	98.77	1.23	
	31.25	0.665	0.011	98.57	1.43

### Dyes decolorization by AgNPs

**Fig. 11:**
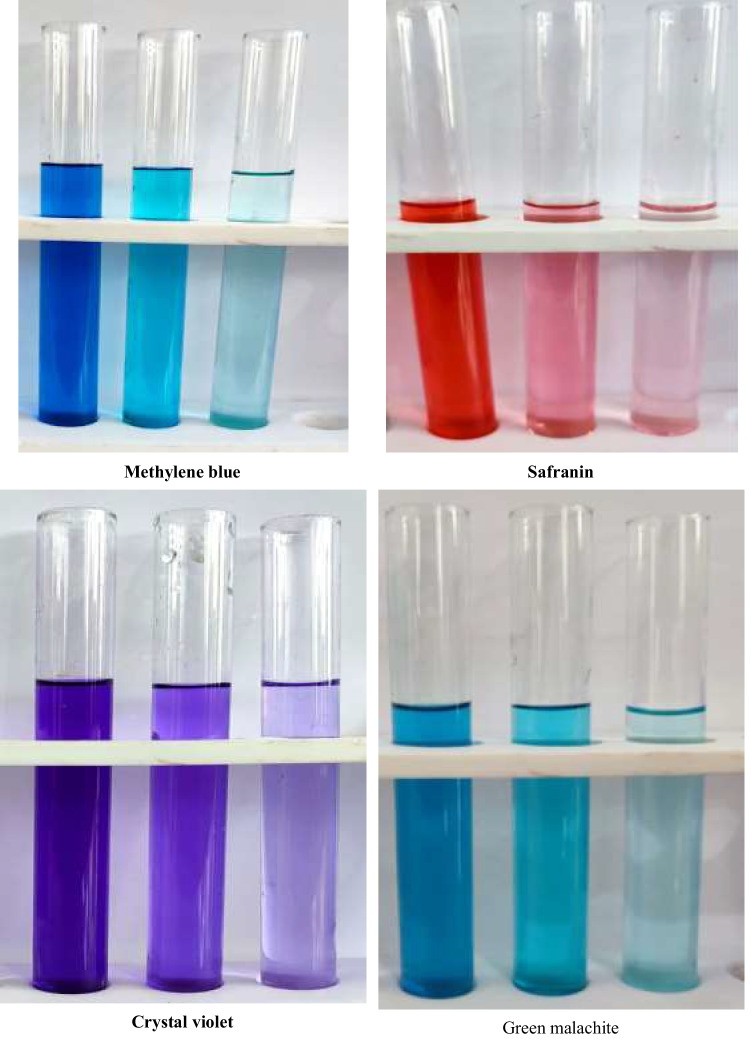
Effect of AgNPs produced from *F. nygamai* isolate AJTYC1 on dyes decolorization

At a concentration of 100 µg/mL, four dyes (methylene blue, safranin, crystal violet, and green malachite) became discolored using the AgNPs produced by *F. nygamai*. According to Table 5 and Fig. 11, the decolorization percent of four dyes increased gradually over time till at 240 min. After 240 min of incubation, the results revealed that the decolorization percent of the methylene blue, safranin, crystal violet, and green malachite dyes by AgNPs were 88.3%, 81.5%, 76.4%, and 78.2%, respectively.

**Table 5:** Effect of different concentrations of AgNPs produced from *F. nygamai* isolate AJTYC1 on dye removal percentages

Time (minutes)	Dye removal (%)			
	Methylene blue	Safranin	Crystal violet	Green malachite
Zero	0	0	0	0
30	22.5 ± 0.1^g^	20.6 ± 0.2^g^	18.9 ± 0.1^g^	20.0 ± 0.2^g^
60	35.6 ± 0.2^f^	30.8 ± 0.2^f^	27.5 ± 0.1^f^	28.8 ± 0.2^f^
0	45.6 ± 0.2^e^	42.3 ± 0.3^e^	39.3 ± 0.2^e^	40.5 ± 0.2^e^
20	60.3 ± 0.3^d^	55.9 ± 0.4^d^	51.0 ± 0.3^d^	52.3 ± 0.4^d^
150	65.3 ± 0.3^c^	62.3 ± 0.5^c^	58.5 ± 0.2^c^	60.1 ± 0.4^c^
180	70.2 ± 0.5^bc^	66.4 ± 0.5^c^	61.7 ± 0.4^c^	63.5 ± 0.5^c^
210	75.4 ± 0.5^b^	72.3 ± 0.4^b^	68.8 ± 0.4^b^	70.0 ± 0.5^b^
240	88.3 ± 0.6^a^	81.5 ± 0.7^a^	76.4 ± 0.5^a^	78.2 ± 0.6^a^

The values are the means of three replicates with standard deviation (± SD). Values followed by the same letter(s) are not significantly different according to Duncan’s multiple range test at *P* ≤ 0.05 level

### Mitotic index

**Fig. 12:**
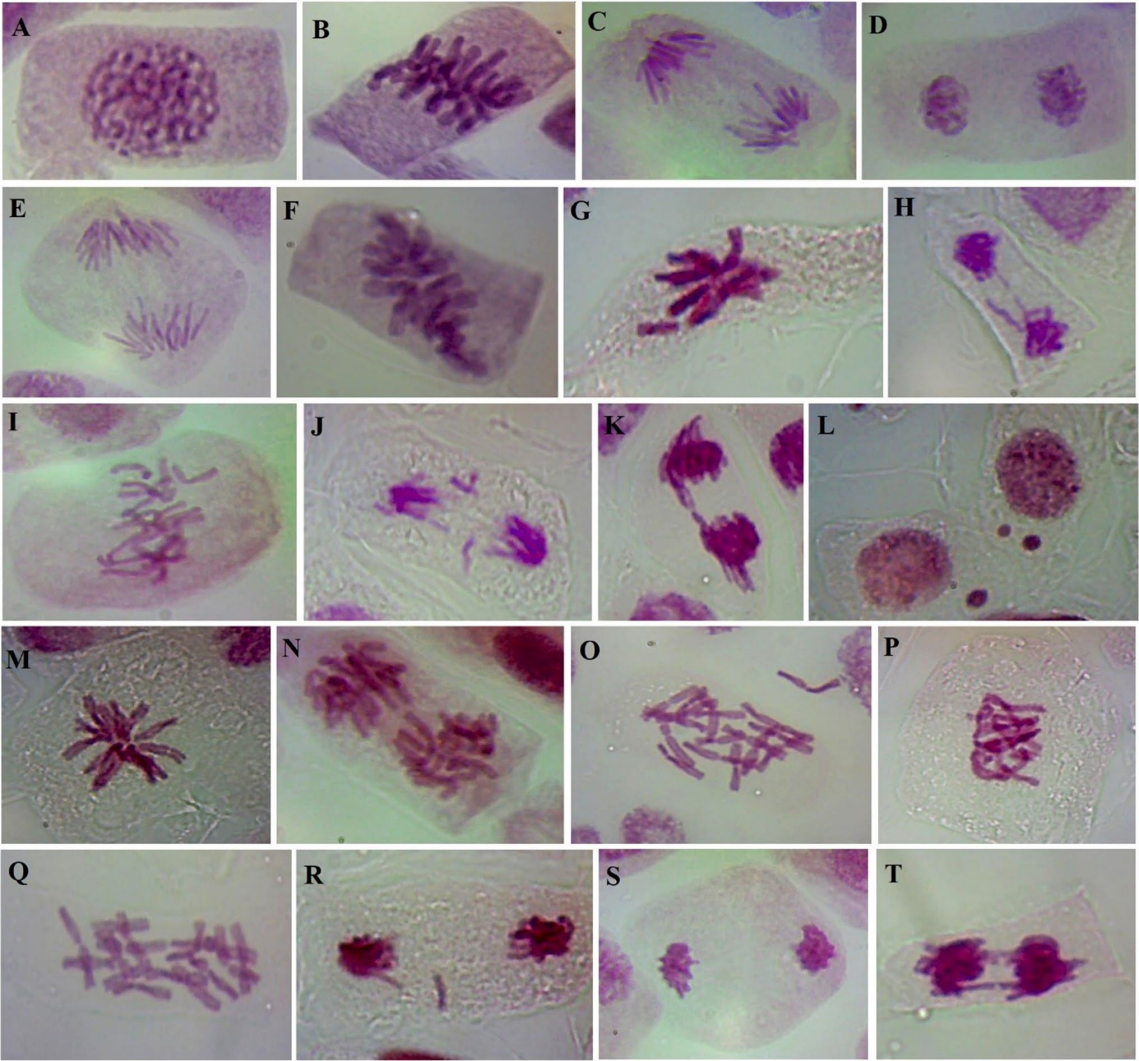
Representative examples of normal and abnormal cell divisions in *Allium cepa* root tips exposed to different treatments. **A**–**D** Normal prophase, metaphase, anaphase, and telophase, respectively, under control conditions (distilled water). **E**, **F** Diagonal phases after treatment with AgNPs (10 mg/L or 6 h). **G** Metaphase with chromosome loss after treatment with AgNPs (20 mg/L for 3 h). **H** Lagging chromosome and chromatin bridge in telophase after treatment with AgNPs (20 mg/L for 12 h). **I** Disturbance in metaphase after treatment with AgNPs (10 mg/L for 12 h). **J** Fragments and lagging chromosomes in anaphase after treatment with AgNPs (30 mg/L or 3 h). **K** Chromosomal bridge in sticky anaphase after treatment with AgNPs (30 mg/L for 6 h). **L** Micronuclei in interphase after treatment with AgNPs (20 mg/L for 12 h). **M** Star metaphase after treatment with AgNPs (20 mg/L for 6 h). **N** Disturbance in anaphase after treatment with AgNPs (20 mg/L for 3 h). **O** C-metaphase after treatment with AgNPs (20 mg/L for 12 h). **P** Sticky metaphase after treatment with AgNPs (30 mg/L for 12 h). **Q** C-metaphase after treatment with AgNPs (30 mg/L for 6 h). **R** Sticky anaphase with lagging chromosome after treatment with AgNPs (30 mg/L for 6 h). **S** Diagonal telophase after treatment with AgNPs (10 mg/L for 12 h). **T** Multibridges in sticky anaphase after treatment with AgNPs (30 mg/L for 12 h)

The data in Table 6 and Fig. 12 show the effect of AgNP concentrations and different exposure times on cell division and chromosomal behavior of *Allium cepa* root tips. According to the current study, AgNPs caused the formation of chromosomal abnormalities as compared to the control group, which had no chromosomal abnormalities. After 3, 6, and 12 h of exposure times, the results showed that the mitotic index value for the control was 11.28, 11.48, and 12.16%. As AgNP concentrations and exposure times increased, the mitotic index decreased in comparison to controls. In comparison to the control, the highest concentrations of AgNPs (30 mg/L) showed the most severe reductions in mitotic index (5.82). Additionally, 12 h after exposure to AgNPs, the most significant reductions in mitotic index were found.

**Table 6:** Mitotic index, phase indices, types, and percentages of mitotic abnormalities (mean ± SD) induced by different concentrations of AgNPs in *Allium cepa* root tips after different exposure times

Treatment		No. of dividing cells	Mitotic index (%)	Phase index (%)				Mitotic aberrations (%)			Total mitotic abnormalities (%)
AgNPs conc. (mg/L)	Exposure time			Prophase	Metaphase	Anaphase	Telophase	Spindle disturbance	Clastogenic aberrations	Chromosome stickiness	
Control	3 h	564	11.28 ± 0.49^a^	50.18 ± 1.77	22.34 ± 0.18	14.54 ± 0.18	12.94 ± 0.18	–	–	–	–
10		479	9.58 ± 0.35^b^	50.52 ± 2.12	22.76 ± 0.71	13.98 ± 0.67	12.73 ± 0.71	6.34 ± 0.35	0.98 ± 0.03	1.42 ± 0.02	8.74 ± 0.41
20		446	8.92 ± 0.33^bc^	52.24 ± 2.26	23.77 ± 0.67	12.78 ± 0.12	11.21 ± 0.63	8.29 ± 0.42	0.46 ± 0.05	1.39 ± 0.07	10.14 ± 0.54
30		408	8.16 ± 0.39^c^	53.43 ± 2.08	25.00 ± 0.52	11.52 ± 0.32	10.05 ± 0.51	8.51 ± 0.65	1.05 ± 0.02	1.47 ± 0.1	11.03 ± 0.76
Control	6 h	574	11.48 ± 0.86^a^	53.31 ± 1.94	21.78 ± 0.35	13.76 ± 0.35	11.15 ± 0.18	–	–	–	–
10		413	8.26 ± 0.42^c^	54.72 ± 2.83	25.91 ± 1.41	11.38 ± 0.71	7.99 ± 0.12	6.45 ± 0.48	1.83 ± 0.07	1.48 ± 0.09	9.76 ± 0.64
20		392	7.84 ± 0.38^c^	53.32 ± 2.37	29.85 ± 0.70	9.18 ± 0.13	7.65 ± 0.66	9.72 ± 0.18	1.89 ± 0.11	2.57 ± 0.14	14.18 ± 0.43
30		336	6.72 ± 0.54^d^	54.46 ± 2.38	30.06 ± 0.82	8.63 ± 0.40	6.85 ± 0.32	10.35 ± 0.22	2.88 ± 0.16	4.98 ± 0.18	18.21 ± 0.56
Control	12 h	608	12.16 ± 0.78^a^	49.34 ± 2.47	23.03 ± 0.18	15.13 ± 0.16	12.50 ± 0.14	–	–	–	–
10		366	7.32 ± 0.28^cd^	55.46 ± 2.12	26.78 ± 0.12	10.38 ± 0.71	7.38 ± 0.61	8.94 ± 0.85	1.92 ± 0.05	1.63 ± 0.08	12.49 ± 0.98
20		346	6.92 ± 0.57^d^	56.07 ± 2.83	28.32 ± 0.82	8.38 ± 0.76	7.23 ± 0.71	10.82 ± 0.41	1.87 ± 0.09	3.24 ± 0.26	15.93 ± 0.76
30		291	5.82 ± 0.52^e^	56.36 ± 2.47	29.21 ± 0.38	8.59 ± 0.53	5.84 ± 0.49	14.82 ± 0.64	4.39 ± 0.12	5.12 ± 0.31	24.33 ± 1.07

5000 cells were scored per treatment

Values followed by the same letter(s) are not significantly different according to Duncan’s multiple range test at *P* ≤ 0.05 level

It is evident from Table 6 and Fig. 12 that the control has no chromosomal abnormality. The total mitotic abnormalities (%) were observed in all AgNPs concentrations and at different periods of exposure. Three major classes of aberrations were distinguished: (a) spindle disturbance and its consequences (e.g., C-metaphase, lagging chromosome, star metaphase/anaphase, multipolar anaphase/telophase, unequal distribution, phase disturbance, and non-oriented chromosome), (b) clastogenic aberrations on chromosomes (e.g., bridges, fragments or breaks, micronuclei, and ring chromosomes), and (c) chromosome stickiness.

The most pronounced increases in total mitotic abnormalities (%) were detected at higher concentrations of AgNPs (30 mg/L) with increasing periods of exposure. The higher mitotic abnormalities (%) were observed after 12 h of exposure to 30 mg/L of AgNPs (24.33%). Different types of chromosomal aberrations were detected in all concentrations of AgNPs and different periods, like diagonal phases, metaphase with chromosome loss, lagging chromosomes and chromatin bridges in telophase, disturbance in metaphase, fragments and lagging chromosomes in anaphase, chromosomal bridges in sticky anaphase, micronuclei in interphase, star metaphase, disturbance in anaphase, C-metaphase, sticky metaphase, C-metaphase, sticky anaphase with lagging chromosomes, diagonal telophase, and multibridges in sticky anaphase, as shown in Fig. 12.

## Discussion

AgNPs can now be produced by green biosynthesis, which has gained popularity as a possible substitute for chemical and physical methods. In addition to stabilizing the generated NPs, metabolites released by *F. nygamai* isolate AJTYC1 are effective in the synthesis of AgNPs (Fig. 1). *Fusarium* spp. are filamentous fungus that can be found in a variety of organic substrates, including soil, water, underground and above-ground plant components, and plant waste. Aqueous silver ions can be reduced extracellularly by the fungus *Fusarium* spp. to produce AgNPs. Both electron shuttle quinones and reductase enzymes are likely involved in this process. Additionally, due to the direct stabilization of the nanoparticles by proteins engaged in the synthesis process, biological nanoparticle synthesis frequently produces a more uniform size distribution pattern than other approaches, as explained by Ishida et al. (2013).

Biogenic AgNPs produced by *F. nygamai* are more desirable and less harmful to the environment than other methods. AgNPs may be produced by proteins or enzymes found in cell-free filtrates of the *F. nygamai* isolate AJTYC1 that convert nitrate to nitrite and then reduced silver ions to metallic silver. The band of AgNPs produced by the *F. nygamai* isolate AJTYC1 cell-free filtrate was apparent as an absorbance peak in the UV–visible spectrum at 420 nm (Fig. 2). AgNPs surface plasmon resonance is responsible for the absorption peak at 400–450 nm, which verifies the synthesis of AgNPs (David and Moldovan 2020). When *F. nygamai* extract was added as a reducing agent to AgNO_3_ solution during the manufacture of AgNPs, a color change from pale yellow to reddish-brown was noticed.

TEM inspection is the most effective method for determining morphological characteristics, such as the size and form of generated AgNPs. The biosynthesized AgNPs had a spherical morphology and ranged in size from 27.3 to 53.1 nm, according to TEM measurements (Fig. 3). In an earlier investigation, the formation of spherical AgNPs with a size range of 6–50 nm was effectively accomplished using TEM according to Alsharif et al. (2020). AgNPs appeared to have a spherical shape in SEM photos. These outcomes are consistent with Zaki et al. (2022).

The XRD pattern of the AgNPs produced by *F. nygamai* was demonstrated using specific peaks in the XRD spectra. XRD-based AgNPs characterization exhibits nine peaks at 2θ values 27.8°, 32.3°, 38.2°, 44.4°, 46.2°, 54.9°, 57.5°, 64.5°, and 77.4° which assigned to planes 012, 200, 110, 202, 220, 116, 222, 400, and 311, respectively, for AgNPs (Fig. 5). Another study found that the face center cubic (fcc) nano-structures of AgNPs (111), (200), (220), (311), and (222) were distinguished by five diffraction peaks at 2θ values of 38.2°, 44.46°, 64.22°, 77.52°, and 81.22°, respectively, with an average size 8–80 nm (Salem et al. 2022). Previous research that demonstrated the creation of AgNPs utilizing microbes had comparable XRD results (Alananbeh et al. 2017).

The zeta potential in this study was discovered to be − 10.79 mV (Fig. 6A). The zeta potential is negative when there is particle repulsion, which raises particle stability. EDX analysis was used to confirm that the filtrate had synthesized AgNPs (Fig. 6B). AgNP concentration was found to be high in this study (70.2%), followed by oxygen (29.8%), indicating that extracellular organic moieties from the filtrate of mycelial free cell were adsorbed on the nanoparticles’ surface (Ahluwalia et al. 2014). By using EDX analysis, it was feasible to confirm that the primary constituent was elemental silver, which was made possible by *Plantago ovata*’s effective conjugation to AgNPs (Githala et al. 2022).

Fourier transform-infrared spectroscopy was used to identify the capping of AgNPs produced by biosynthesis. The probable biomolecules in charge of stabilizing the synthesized silver nanoparticles were found using the FT-IR data. The obvious peaks of the FT-IR data display the values that correspond to the phenolic group (O–H stretching 3433.4 cm^−1^), alcohols and amides groups (O–H stretching, N—H stretch 3194.9 cm^−1^) (Sharma et al. 2023), alkane group (C-H stretching 2926.2 cm^−1^), alkynes group (C = C stretching 2295.5 cm^−1^), cyclic alkenes group (C = C stretching 1634.2 cm^−1^), carboxylic group (C = O 1516.04 cm^−1^), aromatic amine group (C-N stretching 1384.5 cm^−1^), esters group (C—O stretching 1134.3 cm^−1^), and alkene group (C = C bending 824.7 cm^−1^) (Fig. 7).

The formation and capping of AgNPs may be caused by phytochemical components found in *F. nygamai*, including amino acids, phenolic compounds, flavonoids, saponins, glycosides, and carbohydrates, according to FTIR analyses. The phytochemicals from *F. nygamai* that are on the surface of AgNPs may act as a stearic or electrostatic barrier to stop aggregation and stabilize AgNPs (Sharma et al. 2023). It has also been demonstrated that polyphenols have the ability to act as reducing agents in the production of AgNPs. Amide groups are also responsible for the existence of enzymes, and these enzymes are in charge of the reduction synthesis and stabilization of the metal ions (Mekky et al. 2021).

The results showed that AgNPs had superior antioxidant activity when compared to ascorbic acid and butylated hydroxytoluene, which is a powerful antioxidant (Table 3). The functional groups constructively identified through FTIR analysis of the capping agents and the greater surface area and crystalline nature of the NPs (as determined by XRD analysis) may contribute to the improved antioxidant activity's mechanism (Javed et al. 2020). These outcomes are consistent with Gecer et al. (2021) outlined the antioxidant properties of AgNPs produced by *Echinacea purpurea* extract and suggested using AgNPs as food and medicine additives. According to the data, AgNPs have a potential antioxidant activity compared to the standard, butylated hydroxytoluene and ascorbic acid. AgNPs’ effective capacity to scavenge free radicals can be due to the bioactive secondary metabolites found in the filtrate of fungal cells, which serve as a capping agent for nanoparticles. It is interesting that AgNPs have greater H_2_O_2_ and DPPH scavenging activity; this may be because of the way they are designed, characterized, and capped (Govindappa et al. 2018). The reducing power activity was increased with an increase in the AgNPs concentration. The same outcomes are stated by Govindappa et al. (2018) who discovered that the activity of CpAgNPs may be caused by an abundance of phytochemicals on the surface of silver nanoparticles, as they demonstrated 81.64% reducing power test compared to BHT’s (85.64%) (Bhakya et al. 2016).

Today, it is crucial to find or produce safe antimicrobial chemicals in order to combat the issues of antibiotic-resistant bacteria and the growth of microbial infections. Using an agar disc diffusion procedure, the AgNPs synthesized by *F. nygamai* showed effective zone inhibition of the investigated microbes (Table 4 and Fig. 8). AgNPs’ antibacterial efficacy against *S. aureus*, *B. cereus*, *P. aeruginosa*, and *E. coli* was validated by a disc diffusion assay (Chandrasekharan et al. 2022). Another study found that the activity of AgNPs resulted in inhibition zones with varied diameters against *Listeria monocyto*genes (13 mm), *E. coli* (17 mm), *Shigella dysenteriae* (18 mm), and *Salmonella typhi* (14 mm) (Sharma et al. 2022). In contrast to the unicellular fungus *C. albicans*, the data showed that AgNPs had limited antifungal action against the filamentous fungus *A. fumigatus*. These findings were in line with the outcomes of Ahmad et al. (2022), who stated that AgNPs exhibited good antibacterial action, moderate antifungal ability against *Alternaria alternative*, and minimal antifungal potential against *Fusarium gramium*. Also, Al-Rajhi et al. (2022) discovered that AgNPs had excellent inhibitory activity against microorganisms with varied inhibition zones like *B. Subtilis* (28.22 mm), *S. aureus* (23.21 mm), *E. coli* (27.25 mm), *P. aeruginosa* (28.40 mm), *C. albicans* (29.23 mm), and *A. fumigatus* (9.52 mm).

AgNPs may have a broad-spectrum effect because they create irreversible S–Ag bonds with enzymes on cell membranes. As a result, energy production is inhibited, membrane permeability is increased, microbial DNA is interfered with, proteins are disrupted, and ROS are produced, ultimately leading to cell death (Sharma et al. 2022). Also, the disruption of the cell wall caused by the connection of NPs to lipopolysaccharides in the cell membrane facilitates the admission of the NPs into the cell, and the binding of biological elements such as proteins, enzymes, and DNA to the NPs results in cell death (Swamy et al. 2022). Therefore, the interaction between Ag ions and the fungal extract may be responsible for the AgNPs’ reported antibacterial activity as shown in Fig. 9. The electrostatic force of attraction between the less negative to the positive charge of the AgNPs and the negatively charged microbial cell membrane, which results in the conversion of the cells’ chemical and physiological properties, which disrupts the cells’ usual physiological properties such as permeability, respiration, etc., is what causes AgNPs’ significant effect against microbial pathogens (Rudrappa et al. 2023). Additionally, AgNPs have been linked to the production of reactive oxygen and nitrogen species, where they cause oxidative stress on DNA and other cell constituents and disrupt the basic cellular processes in bacterial cells (Dinesh et al. 2022).

The most pronounced effects of AgNPs were detected at a dose of 1000 µg/mL which inhibits tumor viability of MCF-7 (breast cancer), HCT116 (colon cancer), and HepG2 (hepatocellular cancer) by about 2.49%, 5.44%, and 2.67% respectively (Table 5 and Fig. 10). Previous studies have demonstrated that PC-3, lung cancer cell lines, MCF-7, Hep-2, and HeLa cell lines are all susceptible to the cytotoxic effects of biosynthesized AgNPs in vitro (Chen et al. 2022). Even though AgNPs’ anticancer potential has been extensively studied, the activity depends on the AgNPs’ size and where they are produced synthesis. Rajawat et al. (2016) found that 9 nm of AgNPs, as opposed to 15 nm of AgNPs, entirely reduced the proliferation of MCF-7 cancer cell lines. In addition, Erdogan et al. (2019) referred to the mitochondria-mediated caspase apoptosis as one of AgNPs’ inhibitory mechanisms for cancer cell growth. The generation of ROS, followed by the breakdown of intracellular organelles, which eventually results in apoptosis, is how AgNPs carry out their mechanistic function (Seetharaman et al. 2021). Smaller NPs might enter cells without significant endocytosis, spread throughout the cytoplasm, and result in mitochondrial DNA damage and cell death (Rudrappa et al. 2023). AgNP treatment can result in altered cell shape, decreased cell viability, and an increase in lactate dehydrogenase (LDH) production, which causes cell necrosis and apoptosis (Taha et al. 2019).

People and the environment are at risk from the different dyes like methylene blue, safranin, crystal violet, and green malachite. It should definitely be degraded because it is regularly emitted through the effluents of textile manufacturers (Sharma et al. 2023). The results of the photocatalytic degradation of the dyes methylene blue, safranin, crystal violet, and green malachite demonstrate that the percent degradation of different dyes with time is consistent with the results of Jyoti and Singh (2016). Higher specific surface area nanomaterials reportedly supplied more adsorptive active sites for the catalysis process, leading to an improvement in both catalysis and adsorption efficiencies, according to Abdi et al. (2017). Additionally, the presence of O–H bonding seen in the FT-IR analysis may boost ROS production, leading to faster degradation of organic dyes. By producing reactive •OH molecules, which were in charge of oxidizing organic compounds, the hydroxyl groups that support hydroxyl ions made a substantial contribution and caused the breakdown of organic dyes with minimal toxicity (Manikandan et al. 2023).

MB dye was subjected to photocatalytic degradation in the presence of AgNPs (0.2 mg/mL) derived from *Chlorella vulgaris* extract, and it was discovered that 96.51% of the dye had been destroyed after 3 h (Rajkumar et al. 2021). Additionally, Sharma et al. (2023) discovered that the presence of AgNPs accelerated the rate at which MB dye solution faded when exposed to sunlight. According to Thomas and Thalla (2023) study, AgNPs produced using an extract from the seed shells of *Myristica fragrans* showed more than 90% photocatalytic degradation of rhodamine B, methyl violet 10B, and remazol brilliant blue reactive exposed to ultraviolet light.

To assess the genotoxic potential of environmental pollutants, an easy, reliable, and straightforward test model that is simple to administer is the use of onion (*A. cepa*) root tip bioassay (Bakand and Hayes 2016). An essential chromosomal aberration test, such as the *A. cepa* root tip assay, is used for in situ monitoring of environmental pollutants, such as different chemicals and nanoparticles (Debnath et al. 2018).

AgNPs demonstrated cytotoxicity in this study by decreasing the mitotic index, which is similar to the data provided by Debnath et al. (2018). Changes in chromosome structure are referred to as chromosomal aberrations. DNA breakage, the prevention of DNA synthesis, and the replication of changed DNA are only a few of the causes that can result in structural chromosomal abnormalities (Albertini et al. 2000). Chromosome abnormality was confirmed by microscopic analysis that showed lagging chromosomes, stickiness, and anaphase with broken chromosome bridge. Here, several types of chromosomal abnormality were seen together with various salt and nanoparticle concentrations. Here, laggards, broken chromosomal bridges, and anaphase with multiple chromosome bridges were identified alongside clastogenic abnormalities. Other researchers described a similar observation (Debnath et al. 2018).

The decline in the mitotic index (MI) is the most likely effect of AgNPs as seen in Table 6. By inhibiting a significant percentage of cells from entering the prophase, AgNPs may disrupt the normal progression of mitosis and the entire cell cycle (Elghamery et al. 2000). According to Hafez and Fouad (2020), AgNPs exhibit mitoclassic, mitodepressive, and clastogenic capabilities, which can (1) change the orientation of mitotic spindles and interact with the SH of tubulin to cause various spindle disruptions; (2) the chromatin fibers may intertwine, creating stickiness that connects the chromosomes; and (3) there may be fractures that result in the loss of some chromosomal segments. The shifting of poles caused by spindle fibers depolymerization during metaphase and anaphase is the cause of many chromosomal disorders (Hafez and Fouad 2020). The data of the present study at 20 and 30 mg/L of AgNPs was similar to the data of Debnath et al. (2018) at 5 mg/L AgNPs. The failure of anaphase separation was followed by the creation of chromosomal bridges and fragmentations, which are both attributed to chromosomal stickiness (Panda et al. 2011). According to certain studies, in the endosperm cells of plants, acentric chromosome segments are dragged poleward during the creation of the phragmoplast formation by kinetochore independent process which may be one of the reason for anaphase separation (Debnath et al. 2018).

The AgNP may bind to DNA and proteins and change their physico-chemical characteristics, causing harmful changes in their chromatin structure, nucleus condensation, or the formation of inter- and intra-chromatid cross-links. This could explain chromosomal stickiness and other chromosomal abnormalities observed in this study. This theory is supported by (i) the high affinity of AgNPs for DNA, (ii) the synthesis of AgNPs complexes with the ring N atom or NH site in nucleobases of DNA, and (iii) the interaction as well as the establishment of a bio-conjugate between protein and AgNPs (Govindappa et al. 2020).

## Conclusions

In this study, *F. nygamai* isolate AJTYC1 extract was used for the first time to synthesize AgNPs utilizing a simple, effective, and green method. By using TEM, FT-IR, X-ray, and zeta studies, the size, shape, and structure of the biosynthesized NPs were evaluated. The capping activity on the surface of AgNPs is supported by the extract’s polyphenols stretching vibrations. The AgNPs were found at 310 nm. The biosynthesized AgNPs exhibited antioxidant, anticancer, antibacterial, and antifungal properties. AgNPs were shown to be quite effective at removing dye. The results showed that AgNPs had superior antioxidant activity when compared to ascorbic acid and butylated hydroxytoluene, which is a powerful antioxidant. The most pronounced effects of AgNPs were detected at a dose of 1000 µg/mL which inhibits tumor viability of MCF-7 (breast cancer), HCT116 (colon cancer), and HepG2 (hepatocellular cancer). At a concentration of 100 µg/mL, four dyes (methylene blue, safranin, crystal violet, and green malachite) became discolored using the AgNPs. AgNPs caused the formation of chromosomal abnormalities as compared to the control group, which had no chromosomal abnormalities. As AgNP concentrations and exposure times increased, the mitotic index decreased in comparison to controls. In comparison to the control, the highest concentrations of AgNPs (30 mg/L) showed the most severe reductions in mitotic index.

## Data Availability

Not applicable.

## References

[CR1] Abdi J, Vossoughi M, Mahmoodi NM, Alemzadeh I (2017). Synthesis of metal-organic framework hybrid nanocomposites based on GO and CNT with high adsorption capacity for dye removal. Chem Eng J.

[CR2] Ahluwalia V, Kumar J, Sisodia R, Shakil NA, Walia S (2014). Green synthesis of silver nanoparticles by *Trichoderma harzi*anum and their bio-efficacy evaluation against *Staphylococcus aureus* and *Klebsiella pneumonia*. Ind Crop Prod.

[CR3] Ahmad N, Jabeen M, Haq ZU, Ahmad I, Wahab A, Islam ZU, Ullah R, Bari A, Abdel-Daim MM, El-Demerdash FM, Khan MY (2022). Green fabrication of silver nanoparticles using *Euphorbia serpens* Kunth aqueous extract, their characterization, and investigation of its in vitro antioxidative, antimicrobial, insecticidal, and cytotoxic activities. Bio Med Int.

[CR4] Alananbeh K, Al-Refaee W, Al-Qodah Z (2017). Antifungal effect of silver nanoparticles on selected fungi isolated from raw and waste water. Indian J Pharm Sci.

[CR5] Albertini RJ, Anderson D, Douglas GR, Hagmar L, Hemminki K, Merlo F, Natarajan AT, Norppa H, Shuker DE, Tice R, Waters MD (2000). IPCS guidelines for the monitoring of genotoxic effects of carcinogens in humans. Mutat Res/Rev Mutat Res.

[CR6] Alduraihem NS, Bhat RS, Al-Zahrani SA, Elnagar DM, Alobaid HM, Daghestani MH (2023). Anticancer and antimicrobial activity of silver nanoparticles synthesized from pods of *Acacia nilotica*. Processes.

[CR7] Almalki MA, Khalifa AY (2020). Silver nanoparticles synthesis from *Bacillus* sp KFU36 and its anticancer effect in breast cancer MCF-7 cells via induction of apoptotic mechanism. J Photochem Photobiol B.

[CR8] Al-Rajhi AM, Salem SS, Alharbi AA, Abdelghany TM (2022). Ecofriendly synthesis of silver nanoparticles using Kei-apple (*Dovyalis caffra*) fruit and their efficacy against cancer cells and clinical pathogenic microorganisms. Arab J Chem.

[CR9] Alsharif SM, Salem SS, Abdel-Rahman MA, Fouda A, Eid AM, Hassan SED, Awad MA, Mohamed AA (2020). Multifunctional properties of spherical silver nanoparticles fabricated by different microbial taxa. Heliyon.

[CR10] Amaladhas TP, Sivagami S, Devi TA, Nanthi N, Velammal SP (2012). Biogenic synthesis of silver nanoparticles by leaf extract of *Cassia angustifolia*. Adv Nat Sci Nanosci Nanotech.

[CR11] Ameen F, Al-Homaidan AA, Al-Sabri A, Almansob A, AlNAdhari S (2023). Anti-oxidant, anti-fungal and cytotoxic effects of silver nanoparticles synthesized using marine fungus *Cladosporium halotolerans*. App Nanosci.

[CR12] Bakand S, Hayes A (2016). Toxicological considerations, toxicity assessment, and risk management of inhaled nanoparticles. Int J Mol Sci.

[CR13] Bapat MS, Singh H, Shukla SK, Singh PP, Vo DVN, Yadav A, Goyal A, Sharma A, Kumar D (2022). Evaluating green silver nanoparticles as prospective biopesticides: an environmental standpoint. Chemosph.

[CR14] Baskar R, Lee KA, Yeo R, Yeoh KW (2012). Cancer and radiation therapy: current advances and future directions. Int J Med Sci.

[CR15] Bhakya S, Muthukrishnan S, Sukumaran M, Muthukumar M (2016). Biogenic synthesis of silver nanoparticles and their antioxidant and antibacterial activity. App Nano Sci.

[CR16] Bonigala B, Kasukurthi B, Konduri VV, Mangamuri UK, Gorrepati R, Poda S (2018). Green synthesis of silver and gold nanoparticles using *Stemona tuberosa* Lour and screening for their catalytic activity in the degradation of toxic chemicals. Environ Sci Pollut Res.

[CR17] Casagrande MG, Lima RD (2019). Synthesis of silver nanoparticles mediated by fungi: a review. Front Bioeng Biotech.

[CR18] Chandrasekhar N, Vinay SP (2017). Yellow colored blooms of *Argemone mexicana* and *Turnera ulmifolia* mediated synthesis of silver nanoparticles and study of their antibacterial and antioxidant activity. Appl Nano Sci.

[CR19] Chandrasekharan S, Chinnasamy G, Bhatnagar S (2022). Sustainable phyto-fabrication of silver nanoparticles using *Gmelina arborea* exhibit antimicrobial and biofilm inhibition activity. Sci Rep.

[CR20] Chen F, Zheng Q, Li X (2022) *Citrus sinensis* leaf aqueous extract green-synthesized silver nanoparticles: characterization and cytotoxicity, antioxidant, and anti-human lung carcinoma effects. Arabian J Chem 15. 10.1016/j.arabjc.2022.103845103845

[CR21] David L, Moldovan B (2020). Green synthesis of biogenic silver nanoparticles for efficient catalytic removal of harmful organic dyes. Nanomat.

[CR22] Debnath P, Mondal A, Hajra A, Das C, Mondal NK (2018). Cytogenetic effects of silver and gold nanoparticles on *Allium cepa* roots. J Genet Eng Biotechnol.

[CR23] Dhawi F, El-Beltagi HS, Abdel-Mobdy YE, Salah SM, Ghaly IS, Abdel-Rahim EA, Mohamed HI, Soliman AM (2021). Synergistic impact of the pomegranate peels and its nanoparticles against the infection of tobacco mosaic virus (TMV). Fresenius Environ Bulletin.

[CR24] Dimitrijevic R, Cvetkovic O, Miodragović Z, Simit D, Jovik MV (2013). SEM/EDX and XRD characterization of silver nanocrystalline thin film prepared from organometallic solution precursor. J Min Metall Sect B Metall.

[CR25] Dinesh B, Monisha N, Shalini HR, Prathap GK, Poyya J, Shantaram M, Hampapura JS, Karigar CS, Joshi CG (2022). Antibacterial activity of silver nanoparticles synthesized using endophytic fungus *Penicillium cinnamopurpureum*. Spectroscopy Lett.

[CR26] Eisa WH, Zayed MF, Anis B, Abbas LM, Ali SSM, Mostafa AM (2019). Clean production of powdery silver nanoparticles using *Zingiber officinale*: the structural and catalytic properties. J Clean Prod.

[CR27] El-Beltagi HS, El-Mahdy OM, Mohamed HI, El-Ansary AE (2022). Antioxidants, antimicrobial, and anticancer activities of purified chitinase of *Talaromyces funiculosus* strain CBS 129594 biosynthesized using crustacean bio-wastes. Agronomy.

[CR28] Elghamery AA, Elnahas AI, Mansour MM (2000). The action of atrazine herbicide as an inhibitor of cell division on chromosomes and nucleic acids content in root meristems of *Allium cepa* and *Vicia faba*. Cytologia.

[CR29] Erdogan O, Abbak M, Demirbolat GM (2019). Green synthesis of silver nanoparticles via *Cynara scolymus* leaf extracts: the characterization, anticancer potential with photodynamic therapy in MCF7 cells. PLoS One.

[CR30] Gecer EN, Erenler R, Temiz C (2021). Green synthesis of silver nanoparticles from *Echinacea purpurea* (L.) Moench with antioxidant profile. Part Sci Technol.

[CR31] Githala CK, Raj S, Dhaka A, Mali SC, Trivedi R (2022). Phyto-fabrication of silver nanoparticles and their catalytic dye degradation and antifungal efficacy. Front Chem.

[CR32] Gomathi AC, Rajarathinam SX, Sadiq AM, Rajeshkumar S (2020). Anticancer activity of silver nanoparticles synthesized using aqueous fruit shell extract of *Tamarindus Indica* on MCF-7 human breast cancer cell line. J Drug Deliv Sci Technol.

[CR33] Govindappa M, Hemashekhar B, Arthikala MK, Rai VR, Ramachandra YL (2018). Characterization, antibacterial, antioxidant, antidiabetic, anti-inflammatory and antityrosinase activity of green synthesized silver nanoparticles using *Calophyllum tomentosum* leaves extract. Results Physics.

[CR34] Govindappa M, Lavanya M, Aishwarya P, Pai K, Lunked P, Hemashekhar B, Arpitha BM, Ramachandra YL, Raghavendra VB (2020). Synthesis and characterization of endophytic fungi, *Cladosporium perangustum* mediated silver nanoparticles and their antioxidant, anticancer and nano-toxicological study. Bio NanoSci.

[CR35] Guliger-Casagrande M, Germano-Costa T, Pasquoto-Stigliani T, Fraceto LF, Lima R (2019). Biosynthesis of silver nanoparticles employing *Trichoderma harzianum* with enzymatic stimulation for the control of *Sclerotinia sclerotiorum*. Sci Rep.

[CR36] Gurunathan S, Han JW, Eppakayala V, Jeyaraj M, Kim J (2013). Cytotoxicity of biologically synthesized silver nanoparticles in MDA-MB-231 human breast cancer cells. Bio Med Res Int.

[CR37] Hafez RM, Fouad AS (2020). Mitigation of genotoxic and cytotoxic effects of silver nanoparticles on onion root tips using some antioxidant scavengers. Egypt J Bot.

[CR38] Haris M, Hussain T, Mohamed HI, Khan A, Ansari MS, Tauseef A, Khan AA, Akhtar N (2023). Nanotechnology–a new frontier of nano-farming in agricultural and food production and its development. Sci Total Environ.

[CR39] Hublikar LV, Ganachari SV, Patil VB, Nandi S, Honnad A (2023). Anticancer potential of biologically synthesized silver nanoparticles using Lantana camara leaf extract. Prog Biomater.

[CR40] Ihsan M, Din IU, Alam K, Munir I, Mohamed HI, Khan F (2023). Green fabrication, characterization of zinc oxide nanoparticles using plant extract of *Momordica charantia* and *Curcuma zedoaria* and their antibacterial and antioxidant activities. Appl Biochem Biotechnol.

[CR41] Ingle A, Rai M, Gade A, Bawaskar M (2009). *Fusarium solani*: a novel biological agent for the extracellular synthesis of silver nanoparticles. J Nanopart Res.

[CR42] Ishida K, Cipriano TF, Rocha GM, Weissmüller G, Gomes F, Miranda K, Rozental S (2013). Silver nanoparticle production by the fungus *Fusarium oxysporum*: nanoparticle characterisation and analysis of antifungal activity against pathogenic yeasts. Mem Inst Oswaldo Cruz.

[CR43] Javed R, Zia M, Naz S, Aisida SO, Ain Nu, Ao Q (2020). Role of capping agents in the application of nanoparticles in biomedicine and environmental remediation: recent trends and future prospects. J Nano Biotechnol.

[CR44] Jyoti K, Singh A (2016). Green synthesis of nanostructured silver particles and their catalytic application in dye degradation. J Genet Eng Biotechnol.

[CR45] Khodeer DM, Nasr AM, Swidan SA, Shabayek S, Khinkar RM, Aldurdunji MM, Ramadan MA, Badr JM (2023). Characterization, antibacterial, antioxidant, antidiabetic, and anti-inflammatory activities of green synthesized silver nanoparticles using Phragmanthera austroarabica A. G. Mill and J. A. Nyberg extract. Front Microbiol.

[CR46] Kumar G, Ghosh M, Pandey DM (2019). Method development for optimised green synthesis of gold nanoparticles from *Millettia pinnata* and their activity in non-small cell lung cancer cell lines, IET. Nanobiotechnol.

[CR47] Li X, Lin J, Gao Y, Han W, Chen D (2012). Antioxidant activity and mechanism of *Rhizoma Cimicifugae*. Chem Cent J.

[CR48] Manikandan DB, Arumugam M, Sridhar A, Perumalsamy B, Ramasamy T (2023). Sustainable fabrication of hybrid silver-copper nanocomposites (Ag-CuO NCs) using Ocimum americanum L. as an effective regime against antibacterial, anticancer, photocatalytic dye degradation and microalgae toxicity. Environ Res.

[CR49] Meena RK, Meena R, Arya DK, Jadoun S, Hada R, Kumari R (2020). Synthesis of silver nanoparticles by *Phyllanthus emblica* plant extract and their antibacterial activity. Material Sci Res India.

[CR50] Mekky AE, Farrag AA, Hmed AA, Sofy AR (2021). Antibacterial and antifungal activity of green-synthesized silver nanoparticles using *Spinacia oleracea* leaves extract. Egypt J Chem.

[CR51] Mogazy AM, Mohamed HI, El-Mahdy OM (2022). Calcium and iron nanoparticles: a positive modulator of innate immune responses in strawberry plants against *Botrytis cinerea*. Proc Biochem.

[CR52] Moghaddam AB, Namvar F, Moniri M, Tahir PM, Azizi S, Mohamad R (2015). Nanoparticles biosynthesized by fungi and yeast: a review of their preparation, properties, and medical applications. Molecules.

[CR53] Mohamed HI, Abd-Elsalam KA, Tmam AM, Sofy MR (2021) Silver-based nanomaterials for plant diseases management: today and future perspectives. In Silver Nanomaterials for Agri-Food Applications. Kamel A. Abd-Elsalam (ed.). Elsevier, p 495–526. 10.1016/B978-0-12-823528-7.00031-7

[CR54] Niknezhad SV, Najafpour-Darzi G, Morowvat MH, Ghasemi Y (2018). Exopolysaccharide production of *Pantoea* sp. BCCS 001 GH: physical characterizations, emulsification, and antioxidant activities. Int J Biol Macromol.

[CR55] Panda KK, Achary VM, Krishnaveni R, Padhi BK, Sarangi SN, Sahu SN, Panda BB (2011). In vitro biosynthesis and genotoxicity bioassay of silver nanoparticles using plants. Toxicol Vitro.

[CR56] Pandit C, Roy A, Ghotekar S, Khusro A, Islam MN, Emran TB, Lam SE, Khandaker MU, Bradley DA (2022). Biological agents for synthesis of nanoparticles and their applications. J King Saud Univ Sci.

[CR57] Rai M, Bonde S, Golinsk P, Trzcinska-Wencel J, Gade A, Abd-Elsalam KA, Shende S, Gaikwad S, Ingle AP (2021). *Fusarium* as a novel fungus for the synthesis of nanoparticles: mechanism and applications. J Fungi.

[CR58] Rajawat S, Kurchania R, Rajukumar K (2016). Study of anticancer properties of green silver nanoparticles against MCF-7 breast cancer cell lines. Green Process Synth.

[CR59] Rajkumar R, Ezhumalai G, Gnanadesigan M (2021). A green approach for the synthesis of silver nanoparticles by *Chlorella vulgaris* and its application in photocatalytic dye degradation activity. Environ Technol Innov.

[CR60] Ramalingam B, Khan MMR, Mondal B, Mandal AB, Das SK (2015). Facile synthesis of silver nanoparticles decorated magnetic-chitosan microsphere for efficient removal of dyes and microbial contaminants. ACS Sustain Chem Eng.

[CR61] Ranjani S, Shariq AM, Senthil-Kumar N, Ruckmani K, Hemalatha S (2021). Synthesis, characterization and applications of endophytic fungal nanoparticles. Inorg Nano-Metal Chem.

[CR62] Rudrappa M, Kumar RS, Nagaraja SK, Hiremath H, Gunagambhire PV, Almansour AI, Perumal K, Nayaka S (2023). Myco-nanofabrication of silver nanoparticles by *Penicillium brasilianum* NP5 and their antimicrobial, photoprotective and anticancer effect on MDA-MB-231 breast cancer cell line. Antibio.

[CR63] Salem SS, Ali OM, Reyad AM, Abd-Elsalam KA, Hashem AH (2022). *Pseudomonas indica*-mediated silver nanoparticles: antifungal and antioxidant biogenic tool for suppressing mucormycosis fungi. J Fungi.

[CR64] Seetharaman PK, Chandrasekaran R, Periakaruppan R, Gnanasekar S, Sivaperumal S, Abd-Elsalam KA, Valis M, Kuca K (2021). Functional attributes of myco-synthesized silver nanoparticles from endophytic fungi: a new implication in biomedical applications. Biology.

[CR65] Sharma A, Sagar A, Rana J (2022). Green synthesis of silver nanoparticles and its antibacterial activity using fungus *Talaromyces purpureogenus* isolated from *Taxus baccata* Linn. Micro Nano Syst L.

[CR66] Sharma K, Guleria S, Salaria KH, Majeed A, Sharma N, Pawar KD, Thakur VK, Gupta VK (2023). Photocatalytic and biological properties of silver nanoparticles synthesized using *Callistemon lanceolatus* leaf extract. Ind Crops Prod.

[CR67] Siegel RL, Miller KD, Fuchs HE, Jemal A (2021). Cancer Statistics, 2021. CA Cancer J Clin.

[CR68] Sivasubramanian K, Sabarinathan S, Muruganandham M, Velmurugan P, Arumugam N, Almansour A, Kumar R, Sivakumar S (2023). Antioxidant, antibacterial, and cytotoxicity potential of synthesized silver nanoparticles from the Cassia alata leaf aqueous extract. Green Process Synth.

[CR69] Swamy PS, Bhat MP, Nayaka S (2022). *Amycolatopsis* sp. strain MN235945 mediated biosynthesis of silver nanoparticles: characterization, antimicrobial and anticancer activity against HeLa and MCF-7 cell lines. Indian J Pharm Sci.

[CR70] Taha ZK, Hawar SN, Sulaiman GM (2019). Extracellular biosynthesis of silver nanoparticles from *Penicillium italicum* and its antioxidant, antimicrobial and cytotoxicity activities. Biotechnol Lett.

[CR71] Thomas T, Thalla AK (2023). Synthesis of silver nanoparticles using *Myristica fragrans* seed shell: assessment of antibacterial, antioxidant properties and photocatalytic degradation of dyes. J Environ Chem Eng.

[CR72] Varadavenkatesan T, Selvaraj R, Vinayagam R (2020). Green synthesis of silver nanoparticles using *Thunbergia grandiflora* flower extract and its catalytic action in reduction of Congo red dye. Mater Today Proc.

[CR73] Wilson AP, Masters JRW (2000). Cytotoxicity and viability assays. Animal Cell Culture.

[CR74] Ye S, Liu F, Wang J, Wang H, Zhang M (2012). Antioxidant activities of an exopolysaccharide isolated and purified from marine *Pseudomonas* PF-6. Carbohydr Polym.

[CR75] Zaki SA, Ouf SA, Abd-Elsalam KA, Asran AA, Hassan MM, Kalia A, Albarakaty FM (2022). Trichogenic silver-based nanoparticles for suppression of fungi involved in damping-off of cotton seedlings. Microorganisms.

